# Physical activity moderates the association between white matter hyperintensity burden and cognitive change

**DOI:** 10.3389/fnagi.2022.945645

**Published:** 2022-10-12

**Authors:** Suhang Song, Alexandra M. Gaynor, Yunglin Gazes, Seonjoo Lee, Qianhui Xu, Christian Habeck, Yaakov Stern, Yian Gu

**Affiliations:** ^1^Taub Institute for Research on Alzheimer’s Disease and the Aging Brain, Columbia University, New York, NY, United States; ^2^Department of Health Policy and Management, College of Public Health, University of Georgia, Athens, GA, United States; ^3^Division of Cognitive Neuroscience, Department of Neurology, Columbia University, New York, NY, United States; ^4^Gertrude H. Sergievsky Center, Columbia University, New York, NY, United States; ^5^Department of Psychiatry and Biostatistics, Columbia University, New York, NY, United States; ^6^Mental Health Data Science, New York State Psychiatric Institute, New York, NY, United States; ^7^Department of Epidemiology, Joseph P. Mailman School of Public Health, Columbia University, New York, NY, United States; ^8^Department of Psychiatry, Columbia University, New York, NY, United States

**Keywords:** physical activity, white matter hyperintensity (WMH), cognitive change, cognitive reserve, moderation

## Abstract

**Objective:**

Greater physical activity (PA) could delay cognitive decline, yet the underlying mechanisms remain unclear. White matter hyperintensity (WMH) burden is one of the key brain pathologies that have been shown to predict faster cognitive decline at a late age. One possible pathway is that PA may help maintain cognition by mitigating the detrimental effects of brain pathologies, like WMH, on cognitive change. This study aims to examine whether PA moderates the association between WMH burden and cognitive change.

**Materials and methods:**

This population-based longitudinal study included 198 dementia-free adults aged 20–80 years. Leisure-time physical activity (LTPA) was assessed by a self-reported questionnaire. Occupational physical activity (OPA) was a factor score measuring the physical demands of each job. Total physical activity (TPA) was operationalized as the average of z-scores of LTPA and OPA. Outcome variables included 5-year changes in global cognition and in four reference abilities (fluid reasoning, processing speed, memory, and vocabulary). Multivariable linear regression models were used to estimate the moderation effect of PA on the association between white matter hyperintensities and cognitive change, adjusting for age, sex, education, and baseline cognition.

**Results:**

Over approximately 5 years, global cognition (*p* < 0.001), reasoning (*p* < 0.001), speed (*p* < 0.001), and memory (*p* < 0.05) scores declined, and vocabulary (*p* < 0.001) increased. Higher WMH burden was correlated with more decline in global cognition (Spearman’s rho = –0.229, *p* = 0.001), reasoning (rho = –0.402, *p* < 0.001), and speed (rho = –0.319, *p* < 0.001), and less increase in vocabulary (rho = –0.316, *p* < 0.001). Greater TPA attenuated the association between WMH burden and changes in reasoning (β_TPA^*WMH_ = 0.029, 95% CI = 0.006–0.052, *p* = 0.013), speed (β_TPA^*WMH_ = 0.035, 95% CI = –0.004–0.065, *p* = 0.028), and vocabulary (β_TPA^*WMH_ = 0.034, 95% CI = 0.004–0.065, *p* = 0.029). OPA seemed to be the factor that exerted a stronger moderation on the relationship between WMH burden and cognitive change.

**Conclusion:**

Physical activity may help maintain reasoning, speed, and vocabulary abilities in face of WMH burden. The cognitive reserve potential of PA warrants further examination.

## Introduction

It is estimated that greater physical activity (PA) could prevent about 3% of dementias (Livingston et al., 2017; Ogino et al., 2019; Liang et al., 2020; Dominguez et al., 2021). Previous studies have found that greater PA was associated with better cognitive function (Carvalho et al., 2014; Law et al., 2014) and less cognitive decline (Barnes et al., 2003; Weuve et al., 2004; Sofi et al., 2011; Buchman et al., 2012; Baumgart et al., 2015; Willey et al., 2016; Greene et al., 2019; Reas et al., 2019; Nuzum et al., 2020; Dominguez et al., 2021; Soldan et al., 2021). The underlying mechanisms remain unclear, but multiple pathways might be involved. PA might have beneficial effects on maintaining healthy brain status (Gu et al., 2020), which may contribute to better cognitive performance (Prins et al., 2005; Smith et al., 2008; Silbert et al., 2009; Godin et al., 2010; Inaba et al., 2011; Kantarci et al., 2013; Mortamais et al., 2013; Boyle et al., 2016; Windham et al., 2019; Casaletto et al., 2020a). Alternatively, PA may help maintain cognition or prevent cognitive decline by mitigating the detrimental effects of brain changes on cognition, which is a mechanism of cognitive reserve (CR) (Dik et al., 2003; Shatenstein et al., 2015; Christie et al., 2017; Buchman et al., 2019; Tsai and Chang, 2019; Casaletto et al., 2020a,b).

Several cross-sectional studies have found that individuals with greater PA demonstrated better cognition at a given degree of brain pathology, compared to those with lesser PA (Buchman et al., 2019; Casaletto et al., 2020a,b). Among all neuropathological changes examined in these studies, it seems that PA specifically moderates the association between white matter hyperintensity (WMH) burden and cognition, but not necessarily the associations between other brain changes (e.g., AD pathology, Lewy bodies, and Nigral neuronal loss) and cognition among cognitively intact people (Song et al., 2021). WMHs are white matter lesions, which present as hyperintensities on Magnetic Resonance Imaging (MRI), and reflect cerebral small vessel diseases (Smith et al., 2008; Mortamais et al., 2013; Prins and Scheltens, 2015). Higher WMH burden has been shown to predict more cognitive decline in the general population at a late age (Prins et al., 2005; Smith et al., 2008; Silbert et al., 2009; Godin et al., 2010; Inaba et al., 2011; Kantarci et al., 2013; Mortamais et al., 2013; Tosto et al., 2015; Boyle et al., 2016; Windham et al., 2019; Bangen et al., 2020; Rizvi et al., 2021). Interestingly, some subjects maintain relatively stable cognition over time despite the presence of WMH (Kuller et al., 1998; Debette et al., 2010; Mortamais et al., 2013), which could be attributable to higher CR among these individuals (Dufouil et al., 2003; Brickman et al., 2011; Murray et al., 2011; Vemuri et al., 2011). Modifiable lifestyle activity factors, especially physical activity, have been reported to mitigate the impact of WMH burden on cross-sectional measures of cognition (Casaletto et al., 2020a; Song et al., 2021). However, longitudinal studies have been scarce (Aartsen et al., 2002; Willey et al., 2016), and most of those analyses focused on older adults, despite the fact that both brain pathologies and cognitive decline may begin to occur in middle-age (Aartsen et al., 2002; Moura et al., 2019). Besides, few studies examined a comprehensive PA level, which could be composed of PA not only from leisure time but also from occupational activities.

The current study aimed to examine whether PA moderates the association between WMH burden and cognitive change in a population-based study across the adult lifespan. The hypothesis is that PA may delay the cognitive decline given the degree of WMH burden, and such moderation effects may be stronger in older adults compared to the young.

## Materials and methods

### Participants

Participants were derived from two ongoing studies at Columbia University Irving Medical Center: the Cognitive Reserve study and the Reference Ability Neural Network study (Stern et al., 2014, 2018). Participants aged 20–80 years who were right-handed, English speaking, had no psychiatric or neurological disorders, and normal or corrected-to-normal vision were further evaluated with neuropsychological tests, scanned by MRI, and completed socio-demographic information and physical activity questionnaires. To include cognitively intact adults, those with a Dementia Rating Scale (DRS) score < 130 were excluded. More detailed information was provided in previous reports (Stern et al., 2014; Habeck et al., 2016).

At baseline, 562 participants were enrolled in the study, and 255 participants returned for a follow-up visit after about 5 years from their baseline visit as of January 2020. We excluded one participant who developed Multiple Sclerosis by the follow-up visit, and 307 participants either dropped out or have not yet been seen for follow-up. Among 254 participants who completed both baseline and follow-up visits, 15 participants lacking WMH measure, and 41 missing values of PA were excluded. Hence, the current analyses included 198 dementia-free adults who had repeated neuropsychological assessments. The baseline socio-demographic characteristics of the 198 participants were not different from those who were excluded (Supplementary Table 1). The studies were approved by the Institutional Review Board of the College of Physicians and Surgeons of Columbia University.

### Measures

Outcome variables included an average of 5-year cognitive changes in four reference abilities (RAs): fluid reasoning, processing speed, memory, and vocabulary, as well as global cognition, which was the average of the four RAs. As described in the previous studies from our group (Gazes et al., 2021), based on latent change score models (Supplementary methods), each ability was estimated by six cognitive tasks at both baseline and follow-up visits, including three neuropsychological out-of-scanner tests and three computerized in-scanner tasks (Table 1). The cognitive change scores were calculated as the follow-up scores minus the baseline scores.

**TABLE 1:** Measurements of reference abilities.

	Out-of-scanner tests	In-scanner tasks
Fluid reasoning	• WAIS-III Block Design Task; • WAIS-III Letter–Number Sequencing Test; • WAIS-III Matrix Reasoning Test.	• Paper Folding; • Matrix Reasoning; • Letter Sets.
Processing speed	• WAIS-III Digit Symbol; • Trail Making Test-Part A; • Stroop Color Naming Test.	• Digit Symbol; • Letter Comparison; • Pattern Comparison.
Memory	• SRT Long-term Storage; • SRT Continuous Long-term Retrieval; • SRT Total Words Recalled on Last Trial.	• Logical Memory; • Word Order Recognition; • Paired Associates.
Vocabulary	• WAIS-III Vocabulary; • Wechsler Test of Adult Reading; • American National Adult Reading Test.	• Synonyms; • Antonyms; • Picture Naming.

WAIS-III, Wechsler Adult Intelligence Scale-third edition; SRT, Selective Reminding Task.

The weekly frequency of leisure-time physical activities (LTPA) was collected from a self-reported questionnaire of LTPA at baseline. The activities were categorized into three groups based on the intensity^1^ : vigorous, moderate, and light. A standard Metabolic Equivalent of Task (MET) value was assigned to each LTPA group: 9 standard METs for vigorous, 5 for moderate, and 3 for light activities. A weekly MET-minutes score for each LTPA group was calculated by multiplying frequency, estimated average duration (minutes) and its corresponding standard MET value. The total MET-minutes per week for each participant was then calculated as the sum of the three group-specific MET-minutes. LTPA was operationalized as the z-score of the log10 (LTPA + 1)-transformed total MET-minutes per week. Participants also reported their occupation of the longest duration during their lifetime. Matching the participants’ occupations to the Occupational Information Network (O*NET) Standard Occupational Classification codes, 246 O*NET dimensions for each code were obtained. We further performed principal component analysis on the 246 O*NET dimensions and extracted 9 factors. One of these factors measured the physical demands of each job, and was converted into a z-score to indicate occupational physical activity (OPA) level (Gadermann et al., 2014; Habeck et al., 2019). Total PA (TPA) for each participant was estimated by the average of the z-scores of LTPA and OPA.

All brain images were acquired on Philips Achieva 3T MRI. A Fluid-attenuated inversion recovery (FLAIR) scan was acquired with the following parameters: 11,000 ms repetition time, 2800 ms echo time, 256 × 189 voxels in-plane resolution, 23.0 × 17.96 cm field of view, and 30 slices with slice-thickness/gap of 4/0.5 mm and processed through a fully automatic supervised machine learning technique (Ithapu et al., 2014). This method uses a Randomized Decision Trees algorithm called Random Forest for training of the classifier, which has been shown to be superior to the Support Vector Machine algorithm often used for segmenting WMH. The final segmentation is a probability map in [0, 1], which denotes the likelihood that a given voxel is hyperintense, allowing the calculation of WMH volume for each subject. Processed WMH images were visually checked and corrected if voxels were erroneously identified as WMH by trained individuals according to the guidelines established by FreeSurfer. A T1-weighted structural brain image was additionally acquired for each subject using MPRAGE sequence (TE/TR: 3/6.5 ms; Field of view: 256 mm; Flip angle: 8°; In-plane resolution: 256 × 256 voxels; Slice thickness/gap: 1/0 mm; Slices: 180). Total WMH volume, total brain volume (TBV), intracranial volume (ICV), and mean cortical thickness were extracted from structural T1 scans after parcelation using FreeSurfer v5.1^2^ (Moura et al., 2019). In the analysis, the total WMH volume was log10 (WMH + 1)-transformed. Based on a previous study in our group, the age of 43 years was an inflection point at which the total volume of WMH started to increase with age (Moura et al., 2019). Thus, this study included a stratification analysis by age group (<43 years versus ≥43 years). In order to adjust for head size, both TBV and log-transformed WMH were regressed with ICV, and the residuals of TBV and WMH were used in the moderation analysis.

Besides TBV residuals and mean cortical thickness, covariates also included age, sex, years of education, intelligence quotient (IQ), race/ethnicity, cardiovascular risk index, and follow-up intervals. The National Adult Reading Test-assessed IQ (NARTIQ) was used as a continuous variable, as well as age and years of education. Cardiovascular risk index data were collected from a self-reported medical history questionnaire, including four types of cardiovascular diseases (i.e., hypertension, diabetes mellitus, stroke, and myocardial infarction/congestive heart failure/any other heart disease). Cardiovascular risk index ranged from 0 (never being diagnosed or treated for cardiovascular diseases) to 4 (being diagnosed or treated for four cardiovascular diseases). Sex was dichotomized with male as the reference group. Race/ethnicity was categorized into four groups: non-Hispanic white (as the reference group), non-Hispanic black, other non-Hispanic (Asian, others, and mixed race/ethnicity), and Hispanic. Follow-up interval (years) was calculated as the time interval between the baseline and follow-up visits.

### Statistical analysis

Mean, standard deviation (SD), median, and minimum and maximum values were estimated for continuous variables, and frequency and percent were reported for categorical variables. Normally distributed continuous variables were identified by Shapiro-Wilk *W* test, including education, total brain volume, cortical thickness, and baseline global cognition, speed and memory. *T*-tests for normally distributed continuous variables, Wilcoxon rank-sum test for non-normally distributed continuous variables, and Pearson’s chi-square tests for categorical variables were conducted to compare participants’ characteristics between high and low PA groups. Spearman’s correlations, reflected by rho value, were used to examine the monotonicity between WMH burden and cognitive change. The differences in rho were tested between groups using *z*-tests (Hinkle et al., 1988). Multivariable linear regressions were used to estimate the moderation effect of PA by including the interaction term PA*WMH into the models after adjusting for all covariates. In the exploratory analysis, we repeated analyses separately among participants aged < 43 years and among those aged ≥ 43 years. Two-sided *p* < 0.05 indicated significance, except for *p-*values of interaction terms, which were considered statistically significant at *p* < 0.10 since interaction tests are generally underpowered (Brookes et al., 2004; Vivot et al., 2017; Zhao et al., 2020).

## Results

### Characteristics of study population

Table 2 presents a descriptive summary of participants’ characteristics. Participants aged 20–80 years with a mean WMH burden of 1310.95 [SD = 3154.88, median (min, max) = 79.50 (0, 22039)] mm^3^ at baseline were followed for 4.87 (SD = 0.64) years on average. Over approximately 5 years, global cognition (*p* < 0.001), reasoning (*p* < 0.001), speed (*p* < 0.001), and memory (*p* < 0.05) declined, while vocabulary (*p* < 0.001) increased. Participants in the high TPA group had less decline in reasoning (*p* = 0.030) and more increase in vocabulary (*p* = 0.020) than those in the low TPA group. Participants in the high LTPA group, compared to those in the low LTPA group, had a higher education level (*p* = 0.018), a larger total brain volume (*p* = 0.006), better global cognition (*p* = 0.004), reasoning (*p* = 0.006) and speed (*p* = 0.013) at baseline, and less decline in reasoning (*p* = 0.015) and speed (*p* = 0.025). Participants in the high OPA group were more likely to be male (*p* = 0.01) and had a lower education level (*p* = 0.039), compared to the low OPA group. No differences were observed in WMH burden, other demographic characteristics, and cognition between the low and the high PA groups. OPA and LTPA were not correlated with each other (Spearman rho = –0.101, *p* = 0.158) (Supplementary Figure 1).

**TABLE 2:** Summary of characteristics of study participants (*n* = 198).

		All (*n* = 198)	TPA			LTPA			OPA		
					
			Low (*n* = 99)	High (*n* = 99)	*P*-value	Low (*n* = 99)	High (*n* = 99)	*P*-value	Low (*n* = 99)	High (*n* = 99)	*P*-value
Age, years	mean (SD)	54.38 (16.42)	56.38 (15.71)	52.38 (16.95)	0.104	56.1 (15.49)	52.67 (17.20)	0.224	55.52 (16.88)	53.25 (15.95)	0.211
	median (min, max)	61 (20, 80)	63 (22, 80)	57 (20, 77)		62 (22, 80)	58 (20, 79)		63 (22, 79)	57 (20, 80)	
Follow up interval, years	mean (SD)	4.87 (0.64)	4.90 (0.63)	4.85 (0.66)	0.499	4.95 (0.63)	4.80 (0.65)	0.066	4.88 (0.63)	4.87 (0.66)	0.822
	median (min, max)	5 (4, 7)	5 (4, 7)	5 (4, 7)		5 (4, 7)	5 (4, 7)		5 (4, 7)	5 (4, 7)	
Sex											
Male, *n* (%)		90 (45.45)	45 (45.45)	45 (45.45)	1.000	44 (44.44)	46 (46.46)	0.775	36 (36.36)	54 (54.55)	0.010**
Female, *n* (%)		108 (54.55)	54 (54.55)	54 (54.55)		55 (55.56)	53 (53.54)		63 (63.64)	45 (45.45)	
Education,^d^ years	mean (SD)	16.34 (2.42)	16.30 (2.43)	16.38 (2.41)	0.815	15.94 (2.28)	16.75 (2.49)	0.018*	16.70 (2.62)	15.99 (2.15)	0.039*
	median (min, max)	16 (11, 24)	16 (11, 22)	16 (12, 24)		16 (11, 22)	16 (12, 24)		16 (11, 24)	16 (12, 22)	
NARTIQ	mean (SD)	118.29 (8.05)	118.12 (9.13)	118.47 (6.84)	0.399	117.35 (8.49)	119.24 (7.51)	0.158	118.06 (8.83)	118.53 (7.22)	0.617
	median (min, max)	120.48 (94.16, 130.88)	121.76 (94.16, 129.12)	119.36 (98.00, 130.88)		119.60 (94.16, 129.12)	121.36 (98.00, 130.88)		121.36 (99.36, 129.12)	119.84 (94.16, 130.88)	
Race/ethnicity											
Non-Hispanic White, *n* (%)		119 (60.10)	56 (56.57)	63 (63.64)	0.523	54 (54.55)	65 (65.66)	0.263	57 (57.58)	62 (62.63)	0.515
Non-Hispanic Black, *n* (%)		46 (23.23)	25 (25.25)	21 (21.21)		28 (28.28)	18 (18.18)		22 (22.22)	24 (24.24)	
Others,^a^ *n* (%)		10 (5.05)	4 (4.04)	6 (6.06)		4 (4.04)	6 (6.06)		7 (7.07)	3 (3.03)	
Hispanic, *n* (%)		23 (11.62)	14 (14.14)	9 (9.09)		13 (13.13)	10 (10.10)		13 (13.13)	10 (10.10)	
WMH (baseline), mm^3^	mean (SD)	1310.95 (3154.88)	1313.23 (2839.32)	1308.67 (3456.35)	0.673	1586.33 (3979.69)	1035.57 (2004.23)	0.678	1118.76 (1954.73)	1503.14 (4014.04)	0.853
	median (min, max)	79.5 (0, 22039)	80 (0, 22039)	79 (0, 20192)		117 (0, 22039)	62 (0, 8667)		80 (0, 8593)	79 (0, 22039)	
Cardiovascular risk index^c^	mean (SD)	0.27 (0.53)	0.28 (0.52)	0.25 (0.54)	0.454	0.32 (0.55)	0.22 (0.51)	0.143	0.29 (0.52)	0.24 (0.54)	0.276
	median (min, max)	0 (0, 2)	0 (0, 2)	0 (0, 2)		0 (0, 2)	0 (0, 2)		0 (0, 2)	0 (0, 2)	
Total Brain Volume^c,d^ (baseline), cm^3^	mean (SD)	1005.18 (98.51)	994.52 (110.21)	1015.95 (84.28)	0.127	985.92 (111.87)	1024.24 (79.26)	0.006**	995.33 (103.05)	1015.13 (93.16)	0.159
	median (min, max)	1011.94 (675.66, 1254.86)	1011.39 (675.66, 1254.86)	1014.26 (805.03, 1183.96)		1000.16 (675.66, 1243.99)	1021.98 (821.57, 1254.86)		1004.30 (675.66, 1254.86)	1019.87 (803.42, 1183.96)	
Mean cortical thickness^c,d^ (baseline), mm	mean (SD)	2.54 (0.12)	2.53 (0.11)	2.54 (0.13)	0.401	2.54 (0.11)	2.53 (0.13)	0.773	2.54 (0.11)	2.53 (0.12)	0.607
	median (min, max)	2.53 (2.21, 2.82)	2.52 (2.21, 2.79)	2.55 (2.22, 2.82)		2.53 (2.26, 2.79)	2.54 (2.21, 2.82)		2.53 (2.21, 2.82)	2.52 (2.22, 2.80)	
Baseline cognition											
Global cognition^d^	mean (SD)	0 (0.56)	–0.04 (0.57)	0.04 (0.55)	0.282	–0.11 (0.52)	0.11 (0.59)	0.004**	–0.01 (0.6)	0.01 (0.53)	0.787
	median (min, max)	–0.03 (–1.38, 1.32)	–0.04 (–1.38, 1.19)	0.05 (–1.36, 1.32)		–0.13 (–1.38, 1.19)	0.07 (–1.36, 1.32)		–0.03 (–1.38, 1.26)	–0.03 (–1.38, 1.32)	
Fluid reasoning	mean (SD)	0 (0.71)	–0.04 (0.68)	0.04 (0.74)	0.456	–0.14 (0.68)	0.14 (0.72)	0.006**	0.03 (0.70)	–0.04 (0.72)	0.449
	median (min, max)	–0.01 (–1.63, 1.37)	–0.05 (–1.63, 1.34)	0.07 (–1.51, 1.37)		–0.14 (–1.63, 1.33)	0.15 (–1.51, 1.37)		0 (–1.63, 1.37)	–0.02 (–1.62, 1.33)	
Processing speed^d^	mean (SD)	–0.02 (0.69)	–0.10 (0.66)	0.05 (0.70)	0.121	–0.14 (0.63)	0.10 (0.72)	0.013*	–0.03 (0.72)	–0.01 (0.65)	0.865
	median (min, max)	–0.05 (–1.67, 2.12)	–0.11 (–1.67, 1.66)	0.07 (–1.46, 2.12)		–0.14 (–1.67, 1.66)	0.11 (–1.46, 2.12)		–0.11 (–1.46, 2.12)	0.07 (–1.67, 1.79)	
Memory^d^	mean (SD)	–0.02 (0.84)	–0.03 (0.88)	–0.01 (0.80)	0.875	–0.12 (0.84)	0.08 (0.83)	0.102	–0.04 (0.87)	0 (0.80)	0.771
	median (min, max)	0.01 (–2.21, 1.61)	0.01 (–2.15, 1.54)	–0.01 (–2.21, 1.61)		–0.13 (–2.15, 1.54)	0.13 (–2.21, 1.61)		0.01 (–2.15, 1.46)	–0.01 (–2.21, 1.61)	
Vocabulary	mean (SD)	0.04 (0.77)	–0.01 (0.86)	0.09 (0.67)	0.929	–0.06 (0.81)	0.13 (0.72)	0.093	–0.02 (0.87)	0.09 (0.66)	0.978
	median (min, max)	0.29 (–2.31, 1.04)	0.35 (–2.31, 1.04)	0.25 (–1.77, 0.98)		0.17 (–2.31, 1.04)	0.32 (–1.77, 0.98)		0.34 (–2.31, 1.04)	0.25 (–2.03, 0.98)	
Change of cognition											
Global cognition	mean (SD)	–0.1 (0.26)	–0.13 (0.28)	–0.07 (0.23)	0.058	–0.14 (0.28)	–0.06 (0.23)	0.114	–0.10 (0.27)	–0.10 (0.25)	0.857
	median (min, max)	–0.08 (–1.57, 0.45) *** ^b^	–0.12 (–1.57, 0.45)	–0.03 (–0.78, 0.44)		–0.1 (–1.57, 0.34)	–0.06 (–0.69, 0.45)		–0.08 (–1.57, 0.45)	–0.08 (–0.78, 0.44)	
Fluid reasoning	mean (SD)	–0.15 (0.14)	–0.17 (0.16)	–0.13 (0.12)	0.030*	–0.18 (0.15)	–0.13 (0.14)	0.015*	–0.16 (0.16)	–0.15 (0.13)	0.808
	median (min, max)	–0.14 (–1.15, 0.34) *** ^b^	–0.17 (–1.15, 0.34)	–0.13 (–0.57, 0.19)		–0.16 (–1.15, 0.13)	–0.13 (–0.57, 0.34)		–0.14 (–1.15, 0.34)	–0.14 (–0.57, 0.19)	
Processing speed	mean (SD)	–0.18 (0.19)	–0.20 (0.20)	–0.16 (0.17)	0.064	–0.21 (0.19)	–0.15 (0.19)	0.025*	–0.17 (0.20)	–0.19 (0.18)	0.602
	median (min, max)	–0.17 (–1.22, 0.48) *** ^b^	–0.20 (–1.22, 0.46)	–0.15 (–0.62, 0.48)		–0.2 (–1.22, 0.23)	–0.16 (–0.62, 0.48)		–0.17 (–1.22, 0.46)	–0.17 (–0.62, 0.48)	
Memory	mean (SD)	–0.13 (0.69)	–0.20 (0.73)	–0.05 (0.64)	0.176	–0.20 (0.75)	–0.05 (0.63)	0.327	–0.10 (0.70)	–0.15 (0.69)	0.667
	median (min, max)	–0.07 (–2.54, 1.41) * ^b^	–0.12 (–2.54, 1.41)	0.04 (–1.88, 1.40)		–0.10 (–2.54, 1.20)	0.02 (–1.52, 1.41)		–0.10 (–2.54, 1.41)	–0.03 (–1.88, 1.40)	
Vocabulary	mean (SD)	0.06 (0.19)	0.04 (0.21)	0.08 (0.15)	0.020*	0.04 (0.21)	0.08 (0.15)	0.213	0.04 (0.21)	0.08 (0.16)	0.153
	median (min, max)	0.06 (–1.37, 0.62) *** ^b^	0.02 (–1.37, 0.59)	0.09 (–0.47, 0.62)		0.04 (–1.37, 0.59)	0.07 (–0.47, 0.62)		0.04 (–1.37, 0.54)	0.07 (–0.47, 0.62)	

*p < 0.05; **p < 0.01; ***p < 0.001.

SD, standard deviation; TPA, total physical activity; LTPA, leisure time physical activity; OPA, occupational physical activity; WMH, white matter hyperintensity.

^a^Others included Asian, others, and mixed race/ethnicity.

^b^One sample Wilcoxon test (compared to 0) was used to determine if the cognition was significantly changed over 5 years.

^c^Cardiovascular risk index has seven missing values; total brain volume has one missing value; mean cortical thickness has 12 missing values.

^d^Those variables were normally distributed continuous variables; *p*-values were obtained from *t*-test. The other continuous variables were not normally distributed; *p*-values were obtained from Wilcoxon test.

The dichotomous PA groups were split by median PA. The two levels of LTPA were high (≥1260 MET-min/week) and low (0 to <1260 MET-min/week). People included in the high LTPA group should perform at least 2.33 h of vigorous activities, 4.2 h of moderate activities, or 7 h of light activities per week. In terms of OPA, the people in high OPA group mainly work on jobs with high demands of outdoor and physical activities, such as construction workers, firefighters, police, truck drivers, real estate sales agents, etc. Bold values indicate significant values (*p* < 0.05 or 0.1).

### Correlation between white matter hyperintensity burden and cognition

Higher WMH burden was significantly correlated with more decline in global cognition (rho = –0.229, *p* = 0.001), reasoning (rho = –0.402, *p* < 0.001), and speed (rho = –0.319, *p* < 0.001), and less increase in vocabulary (rho = –0.316, *p* < 0.001) (Table 3). Similar correlations were reported in both low and high TPA groups, with stronger correlations among participants with lesser TPA than those with greater TPA, especially for vocabulary (*p-diff* = 0.010). As for the correlations between WMH burden and cognitive change by LTPA and OPA groups, such correlations seemed to be stronger in the low OPA group and high LTPA group among total participants, but didn’t reach statistical significance (Supplementary Table 2).

**TABLE 3:** Spearman’s correlations between white matter hyperintensity (WMH) and cognitive change within total physical activity groups (TPA) (*n* = 198).

		Total population (*n* = 198)				Aged ≥ 43 years (*n* = 147)				Aged < 43 years (*n* = 51)			
		All	Low TPA (*n* = 99)	High TPA (*n* = 99)	*p*-diff^a^	All	Low TPA (*n* = 77)	High TPA (*n* = 70)	*p*-diff^a^	All	Low TPA (n = 22)	High TPA (n = 29)	*p*-diff^a^
Global cognition change	rho	–0.229**	–0.278**	–0.183	0.487	–0.123	–0.244*	–0.007	0.151	–0.105	–0.118	–0.099	0.949
	*p*	0.001	0.005	0.070		0.138	0.032	0.956		0.464	0.601	0.609	
Fluid reasoning change	rho	–0.402***	–0.486***	–0.324**	0.177	–0.244**	–0.483***	0.002	0.002**	–0.088	–0.033	0.004	0.902
	*p*	<0.001	<0.001	0.001		0.003	<0.001	0.988		0.538	0.883	0.982	
Processing speed change	rho	–0.319***	–0.412***	–0.223*	0.143	–0.190*	–0.419***	0.033	0.005**	–0.013	0.027	0.037	0.974
	*p*	<0.001	<0.001	0.027		0.021	<0.001	0.785		0.925	0.907	0.847	
Memory change	rho	–0.118	–0.135	–0.099	0.800	–0.099	–0.130	–0.071	0.724	–0.099	–0.16	–0.011	0.618
	*p*	0.099	0.182	0.327		0.231	0.259	0.562		0.488	0.477	0.956	
Vocabulary change	rho	–0.316***	–0.474***	–0.142	0.010**	–0.100	–0.306**	0.125	0.009**	–0.126	–0.117	–0.146	0.922
	*p*	<0.001	<0.001	0.162		0.230	0.007	0.303		0.377	0.604	0.450	

*p < 0.05; **p < 0.01; ***p < 0.001.

TPA, total physical activity; WMH, white matter hyperintensity.

^a^*p*-diff values referred to the significance between low- versus high-PA group, which were calculated in MedCalc (Free trial) software: https://www.medcalc.org/. The dichotomous TPA groups were split by median TPA. Bold values indicate significant values (*p* < 0.05 or 0.1).

### Moderation effects of physical activity on the association between white matter hyperintensity burden and cognitive change

After adjusting for age, sex, education, and baseline cognition, there were significant interactions between TPA and WMH burden on changes in reasoning (β_TPA^*WMH_ = 0.029, 95% CI = 0.006–0.052, *p* = 0.013), speed (β_TPA^*WMH_ = 0.035, 95% CI = 0.004–0.065, *p* = 0.028), and vocabulary (β_TPA^*WMH_ = 0.034, 95% CI = 0.004–0.065, *p* = 0.029), indicating that participants with greater TPA showed disproportionately less decline in reasoning and speed and more increase in vocabulary in face of their WMH burden, compared to those with lesser TPA (Table 4). Findings for OPA were aligned with the findings for TPA; LTPA didn’t moderate the relationship between WMH burden and change of cognition. After additionally adjusting for all covariates, similar findings were observed.

**TABLE 4:** The interaction of physical activity (PA) with white matter hyperintensity (WMH) on cognitive change.

		Global cognition change			Fluid reasoning change			Processing speed change			Memory change			Vocabulary change		
				
		Model 1	Model 2	Model 3	Model 1	Model 2	Model 3	Model 1	Model 2	Model 3	Model 1	Model 2	Model 3	Model 1	Model 2	Model 3
Total population																
TPA (zscore)* WMH	Estimate	0.023	0.027	0.029	0.029**	0.028***	0.029***	0.035**	0.035**	0.036**	0.018	0.034	0.033	0.034**	0.035***	0.037***
	95% CI	–0.022–0.068	–0.016–0.070	–0.015–0.073	0.006–0.052	0.007–0.048	0.008–0.051	0.004–0.065	0.005– 0.065	0.006–0.067	–0.100–0.137	–0.086–0.154	–0.091–0.157	0.004–0.065	0.010–0.061	0.010–0.063
	*p*	0.313	0.214	0.191	0.013	0.008	0.007	0.028	0.021	0.021	0.762	0.578	0.600	0.029	0.006	0.007
LTPA (zscore)* WMH	Estimate	0.005	0.005	0.008	0.003	0.001	0.002	0.002	0.001	0.002	0.007	0.011	0.017	0.007	0.007	0.008
	95% CI	–0.030–0.040	–0.028–0.038	–0.026–0.041	–0.015–0.021	–0.015–0.017	–0.014–0.018	–0.022–0.027	–0.022–0.024	–0.022–0.026	–0.085–0.098	–0.081–0.103	–0.076–0.110	–0.017–0.031	–0.013–0.026	–0.012–0.028
	*p*	0.777	0.778	0.657	0.724	0.888	0.801	0.849	0.931	0.856	0.888	0.818	0.723	0.558	0.514	0.446
OPA (zscore)* WMH	Estimate	0.012	0.015	0.015	0.016**	0.016***	0.016***	0.020**	0.021**	0.021**	0.011	0.020	0.015	0.016*	0.017**	0.017**
	95% CI	–0.012–0.037	–0.008–0.038	–0.009–0.039	0.003–0.028	0.005–0.027	0.005–0.028	0.003–0.037	0.005–0.037	0.004–0.038	–0.054–0.077	–0.045–0.085	–0.053–0.083	–0.001–0.033	0.003–0.030	0.002–0.032
	*p*	0.326	0.193	0.226	0.015	0.005	0.006	0.020	0.012	0.016	0.729	0.544	0.666	0.061	0.016	0.022
Aged ≥ 43 years																
TPA (zscore)* WMH	Estimate	0.046	0.045	0.039	0.037***	0.034***	0.035***	0.045**	0.044**	0.040**	0.073	0.074	0.054	0.036*	0.035**	0.039**
	95% CI	–0.012–0.104	–0.009–0.098	–0.017–0.094	0.009–0.065	0.011–0.057	0.010–0.059	0.007–0.082	0.009–0.078	0.004–0.076	–0.078–0.225	–0.079–0.227	–0.105–0.212	–0.003–0.075	0.006–0.064	0.008–0.070
	*p*	0.120	0.103	0.167	0.010	0.004	0.005	0.020	0.014	0.030	0.340	0.341	0.504	0.066	0.019	0.014
LTPA (zscore)* WMH	Estimate	0.017	0.016	0.020	0.008	0.007	0.007	0.005	0.003	0.003	0.001	0.008	0.020	0.011	0.007	0.011
	95% CI	–0.039–0.072	–0.036–0.068	–0.033–0.073	–0.019–0.036	–0.017–0.030	–0.017–0.032	–0.031–0.042	–0.031–0.038	–0.033–0.038	–0.144–0.145	–0.140–0.156	–0.131–0.170	–0.026–0.049	–0.022–0.036	–0.020–0.041
	*p*	0.554	0.542	0.459	0.551	0.571	0.543	0.777	0.847	0.883	0.994	0.916	0.796	0.559	0.622	0.490
OPA (zscore)* WMH	Estimate	0.020	0.020	0.015	0.016**	0.015**	0.015**	0.022**	0.021**	0.020**	0.043	0.041	0.025	0.014	0.014*	0.016*
	95% CI	–0.011–0.050	–0.008–0.047	–0.014–0.044	0.002–0.031	0.003–0.027	0.003–0.028	0.002–0.041	0.003–0.039	0.001–0.038	–0.036–0.122	–0.037–0.119	–0.057–0.108	–0.007–0.034	–0.001–0.029	–0.0004–0.032
	*p*	0.199	0.162	0.309	0.031	0.012	0.017	0.031	0.020	0.040	0.286	0.304	0.546	0.183	0.063	0.055
Aged < 43 years																
TPA (zscore)* WMH	Estimate	–0.053	–0.085	–0.182	–0.052	–0.041	–0.085	–0.075	–0.095	–0.154	0.023	–0.095	–0.266	–0.062	0.008	–0.006
	95% CI	–0.280–0.174	–0.350–0.180	–0.498–0.134	–0.174–0.069	–0.187–0.105	–0.264–0.093	–0.249–0.099	–0.310–0.120	–0.405–0.097	–0.590–0.635	–0.772–0.581	–1.115–0.583	–0.222–0.098	–0.149–0.165	–0.203–0.191
	*p*	0.639	0.520	0.250	0.390	0.574	0.336	0.388	0.377	0.220	0.941	0.777	0.527	0.436	0.921	0.954
LTPA (zscore)* WMH	Estimate	–0.023	0.110	0.165	0.021	0.056	0.070	0.010	0.048	0.059	–0.089	0.165	0.228	0.012	0.042	0.058
	95% CI	–0.225–0.179	–0.126–0.346	–0.104–0.434	–0.088–0.129	–0.072–0.183	–0.073–0.212	–0.144–0.163	–0.133–0.229	–0.142–0.260	–0.628–0.450	–0.439–0.770	–0.479–0.936	–0.127–0.151	–0.103–0.187	–0.108–0.223
	*p*	0.822	0.351	0.220	0.705	0.382	0.327	0.899	0.596	0.553	0.741	0.583	0.515	0.862	0.559	0.482
OPA (zscore)* WMH	Estimate	–0.012	–0.040	–0.074	–0.017	–0.018	–0.034	–0.020	–0.031	–0.047	0.017	–0.044	–0.102	–0.019	–0.004	–0.016
	95% CI	–0.119–0.094	–0.161–0.082	–0.211–0.063	–0.074–0.039	–0.084–0.047	–0.108–0.040	–0.102–0.062	–0.127–0.066	–0.152–0.059	–0.265–0.298	–0.352–0.264	–0.465–0.261	–0.093–0.054	–0.075–0.067	–0.099–0.068
	*p*	0.816	0.511	0.28	0.541	0.575	0.358	0.624	0.523	0.375	0.907	0.774	0.571	0.596	0.909	0.702

*p < 0.1; **p < 0.05; ***p < 0.01.

TPA, total physical activity; LTPA, leisure time physical activity; OPA, occupational physical activity; WMH, white matter hyperintensity; 95% CI, 95% confidence interval.

Covariates: Model 1: age, sex, education, baseline cognition. Model 2: Model 1 + IQ, race/ethnicity (categorical), cardiovascular risk index. Model 3: Model 2 + total brain volume, mean cortical thickness. Bold values indicate significant values (*p* < 0.05 or 0.1).

**FIGURE 1:**
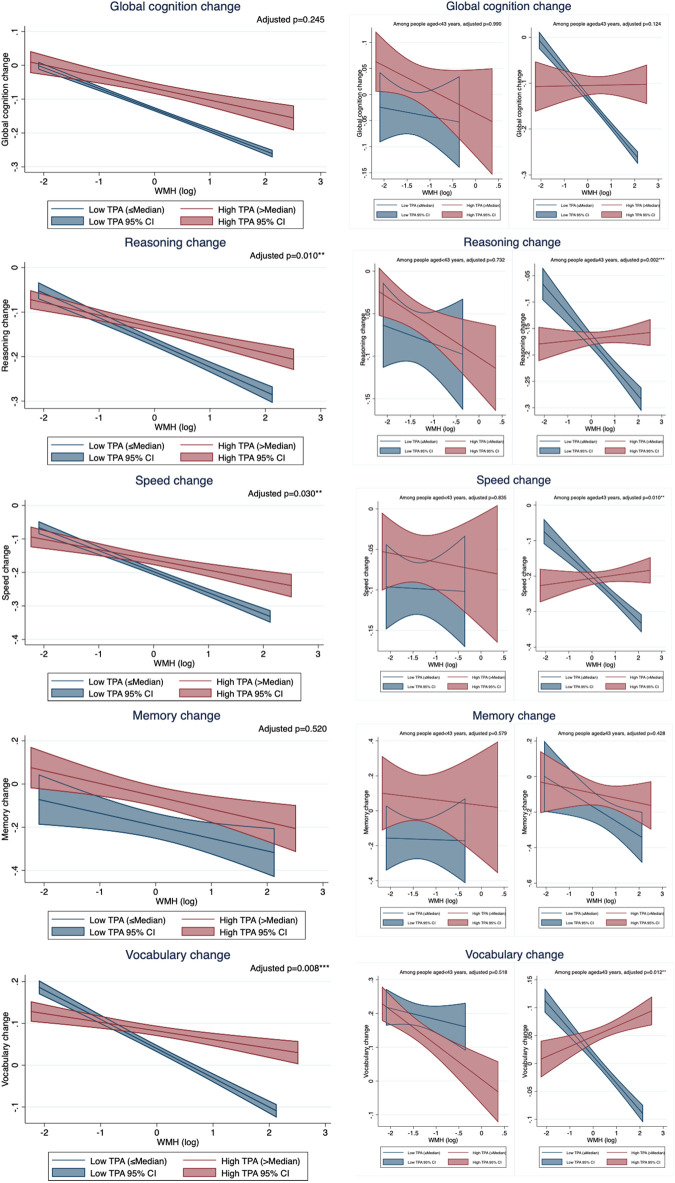
The relationship between white matter hyperintensity (WMH) and cognitive change by total physical activity (TPA) group. TPA, total physical activity; WMH, white matter hyperintensity. All models controlled for age, sex, education, and baseline cognition. Adjusted *p*-values were the *p*-values of the interaction term between WMH and binary TPA group.

We further examined the association between WMH and cognitive change stratified by dichotomous (median-split) PA groups. We found WMH was associated with the decline in reasoning (*p-interaction* = 0.010), speed (*p-interaction* = 0.030), and vocabulary (*p-interaction* = 0.008) to a less extent in those with high TPA than those with low TPA (Figure 1 and Supplementary Table 3). The moderation effects of binary LTPA and OPA didn’t reach significance, except for the moderation effect of OPA on the association between WMH and vocabulary change (*p*-interaction = 0.078) (Supplementary Table 3 and Supplementary Figures 2, 3).

### Exploratory analysis

The exploratory analysis was conducted by age group. Among participants aged ≥ 43 years, higher WMH burden was significantly correlated with more decline in reasoning and speed in all participants, and in those with lesser TPA, but not in those with greater TPA, with a significant difference of correlation coefficients between low and high TPA groups (*p-diff* = 0.002 for reasoning, *p-diff* = 0.005 for speed) (Table 3). Among participants aged < 43 years, WMH burden was not correlated with cognitive change in all participants, or within either TPA subgroup.

Similar to the main findings of moderation effects, TPA and OPA significantly attenuated the relationship between WMH burden and the changes in reasoning, speed and vocabulary in the older group, but not in the younger group (Table 4 and Figure 1). LTPA did not moderate the association between WMH burden and cognitive change in either age group (Table 4).

## Discussion

In this longitudinal study of 198 participants, we found that global cognition, reasoning, speed, and memory scores declined, and vocabulary increased over about 5 years. Higher baseline WMH burden was correlated with more decline in global cognition, reasoning and speed, and less increase in vocabulary. Greater TPA and OPA attenuated the associations between WMH burden and changes in reasoning, speed, and vocabulary.

The findings of the increase in vocabulary (Gold et al., 1995; Schaie, 1996; Ben-David et al., 2015; Gazes et al., 2021) and the decline in other RAs (Ockleford, 2009; Gazes et al., 2021) with increasing age are aligned with previous studies. The explanation of the vocabulary trajectory may lie in the growing opportunities to expose to new words and to use previously learned words, which may further enhance memory traces (Burke et al., 2000; Ben-David et al., 2015). Accordingly, the accumulated life experiences among older people may provide enriched information that may be used as an alternate pathway to better understanding the meaning of a word (dual-representation theory of knowledge) (Brainerd and Reyna, 1992; McGinnis and Zelinski, 2003; Ben-David et al., 2015). As a result, as age increases, older adults perform better in domains that may rely on gathering knowledge from previous experiences, such as vocabulary (MacKay and Burke, 1990; Ben-David et al., 2015). However, when it comes to cognitive domains emphasizing speed or the inhibition of irrelevant information, performance declines with age (Schneider et al., 2010; Ben-David et al., 2012, 2014).

The negative correlation between WMH burden and cognitive change was consistent with other studies (Prins et al., 2005; Smith et al., 2008; Silbert et al., 2009; Godin et al., 2010; Inaba et al., 2011; Kantarci et al., 2013; Mortamais et al., 2013; Boyle et al., 2016; Windham et al., 2019). The primary potential mechanism may be an association between WMH burden and cerebrovascular diseases due to ischemic issues, such as infarction and gliosis, which may reflect an evolving pathologic process (Godin et al., 2010; Boyle et al., 2016). Besides, WMH burden may be related to other neurodegenerative processes. WMH burden together with other comorbid pathologies may result in an earlier onset of cognitive impairments, which may decrease the ability of brain to tolerate the cognitive decline (Boyle et al., 2016; Tanskanen et al., 2017). Moreover, WMH burden may be associated with different processes such as inflammation or other vascular dysfunctions, and hence may impair cognition through other pathways (Rosenberg, 2009; Boyle et al., 2016; Gu et al., 2019).

This study found that PA moderates the associations between WMH burden and reasoning, speed, or vocabulary. This finding is consistent with previous studies which also reported a moderation effect of PA on the associations of WMH burden with cross-sectional global cognition (Casaletto et al., 2020a) and with the decline in speed (Willey et al., 2016). One interpretation could be that greater PA may increase cerebral blood flow (Scarmeas et al., 2003; Dominguez et al., 2021) to assist in addressing possible ischemic issues, which are related to higher WMH burden, so as to mitigate the potential cognitive decline due to WMH burden. Alternatively, active engagement in PA may increase synaptogenesis of the unaffected neurons to compensate for the brain damage (i.e., WMH burden), and hence the brain can tolerate more loss before developing cognitive impairments (Scarmeas and Stern, 2003; Fratiglioni et al., 2004; Scarmeas, 2007).

This study did not find a correlation between WMH burden and memory change, or a moderation effect of PA on such association. The different findings between memory and other RAs are aligned with previous findings, reporting that WMH burden was associated with other cognitive functions, especially speed (Gunning-Dixon and Raz, 2000; Prins et al., 2005; Inaba et al., 2011), but not memory after controlling for confounders (Prins et al., 2005). WMH burden may reduce the speed of neural transmission and inter-neuronal connectivity, resulting in the decline in speed (Gunning-Dixon and Raz, 2000).

The different moderation effects on the relationship between WMH burden and cognitive change by LTPA versus OPA groups indicated that the specific type of PA, such as LTPA, may not capture the entire benefits of PA, and thus it is of great value to assess the impact of the combination of various PA types on cognitive change in face of WMH burden. Consistent with a previous study, LTPA didn’t moderate the association between WMH burden and cognitive change (Willey et al., 2016). Few studies have examined the role of OPA in cognitive change given the degree of WMH burden. However, occupation has been considered as a CR proxy, which may delay the decline of cognition in face of brain markers (Stern et al., 1995). Our study further suggests that OPA may contribute to the occupation, mitigating the association between WMH burden and cognitive change.

Using 43 years of age to categorize the young and older adults was according to an inflection point found by our lab, indicating that the accumulation of WMH burden started in middle age (Moura et al., 2019). However, previous studies examining PA-WMH interaction on cognition have mainly focused on older adults, instead of the adults across the lifespan (Casaletto et al., 2020a). In this study, the moderation effect of PA was observed among people ≥ 43 years, suggesting that PA may provide protection against WMH’s detrimental effect on cognition at an earlier age than the typical age of onset of clinical cognitive impairments or dementias (Debette et al., 2011; Moura et al., 2019). We didn’t find evidence that PA moderates the WMH-cognitive change association in younger adults. In addition to the lesser burden of WMH to fight against, the younger adults may also have had less occupational exposure to physical activities compared to older adults, who might have a longer job history.

This study is subjected to several limitations. First, the LTPA questionnaire collected information on LTPA in the past week during the baseline visits, so it may not represent the habitual long-term LTPA and cannot capture the change in LTPA habits. Second, the assessment of OPA was different from the measurement of LTPA, which may be associated with the different findings by type of PA, although z-score was used to standardize the measurements. Third, this study focused on people living in the New York City metropolitan area; further studies conducted among a more variable population in different areas might be needed. Fourth, the time interval from baseline to follow-up was about 5 years, which may be too short an interval to observe changes in some RAs affected by PA. Fifth, the CR/RANN study is currently ongoing and in the process of completing 5-year follow-up, and many participants are not due for a follow-up visit yet, which makes the sample size relatively small when examining the moderation effects by age groups. Thus, we might miss smaller effects due to the small sample size. Last, our processing pipeline did not specify deep or periventricular WMH burdens. Examining whether PA moderates deep or periventricular WMH burden on cognition would assist in exploring the underlying mechanisms and is warranted in future studies.

This study also has several notable strengths. This study examined the moderating role of PA in the associations between WMH burden and cognitive changes using a longitudinal study design, and is thus less likely to be subject to reverse causality compared to cross-sectional studies. This study analyzed the effects of TPA in addition to LTPA and OPA, which allowed for the evaluation of multiple physical activity situations to provide a more generalized conclusion. The study included adults across the lifespan, and therefore allowed for comparisons of the effect size of PA between young and older population. The study controlled for many potential confounders including brain measures, resulting in more reliable and unbiased findings. Lastly, this study was strengthened by the inclusion of cognitive scores based on 24 cognitive measures using latent change score modeling, resulting in robust estimates of changes in four RAs.

## Conclusion

Physical activity may provide cognitive reserve to maintain the abilities in reasoning, speed, and vocabulary in face of WMH burden in middle-aged and older adults.

## Data availability statement

The data presented in this study are available on request from the corresponding author.

## Ethics statement

The studies involving human participants were reviewed and approved by Institutional Review Board of the College of Physicians and Surgeons of Columbia University. The patients/participants provided their written informed consent to participate in this study.

## Author contributions

YGu and SS: conception or design of the work. SS: drafting of manuscript. All authors contributed to critical revision and final approval of the manuscript and fulfilled the ICMJE criteria for authorship.

## Footnotes

1 Vigorous LTPA includes running, jogging, hockey, football, soccer, squash, basketball, cross country skiing, judo, roller skating, vigorous swimming, and vigorous long-distance bicycling. Moderate LTPA includes fast walking, baseball, tennis, easy bicycling, volleyball, badminton, easy swimming, alpine skiing, and popular and folk dancing. Light LTPA includes yoga, archery, fishing from riverbank, bowling, horseshoes, golf, snowmobiling, and easy golf.

2
http://surfer.nmr.mgh.harvard.edu/
