# Supplementary material for: Physical activity moderates the association between white matter hyperintensity burden and cognitive change
Source: Front Aging Neurosci. 2022 Oct 12;14:945645. doi: 10.3389/fnagi.2022.945645 (PMC9610117; doi:10.3389/fnagi.2022.945645)
Supplement: Supplementary file 1 [file Data_Sheet_1.docx]

Supplementary Materials

[sMethods 2](#_Toc98709702)

[sTable 1. Comparison of the participants’ characteristics between those who were included in the final models and who were not, baseline. 9](#_Toc98709703)

[sTable 2. Spearman’s correlation between white matter hyperintensity (WMH) and cognitive change within leisure time physical activity (LTPA) and occupational physical activity (OPA) groups (n=198). 10](#_Toc98709704)

[sTable 3. The interaction of dichotomous physical activity group with white matter hyperintensity (WMH) on cognitive change. 12](#_Toc98709705)

[sFigure 1. The association between leisure time physical activity and occupational physical activity. 14](#_Toc98709706)

[sFigure 2. The relationship between white matter hyperintensity (WMH) and cognitive change by leisure time physical activity (LTPA) group. 15](#_Toc98709707)

[sFigure 3. The relationship between white matter hyperintensity (WMH) and cognitive change by occupational physical activity (OPA) group. 17](#_Toc98709708)

# sMethods

Latent change score

To quantify changes in the four reference abilities, we used a multiple indicator latent change score model (LCSM) (Kievit et al., 2018) as depicted in Supplementary Method Figure 1. The LCSM models changes in the latent scores rather than the observed scores. We modeled the four RAs in the manner of traditional confirmatory factor analysis as described in previous studies (Salthouse et al., 2015; Stern et al., 2014). The factor loadings at baseline and follow-up were constrained to be the same. The overall fit statistics are summarized in Supplementary Method Table 1.

The full summary of the model fits is shown in Supplementary Method Table2. All analysis was performed in lavvan package version 0.6.7 (Rosseel, 2012). The factor scores were estimated using “lavPredict” function.

References:

1. R. A. Kievit, A. M. Brandmaier, G. Ziegler, et al. Developmental cognitive neuroscience using latent change score models: A tutorial and applications. Developmental Cognitive Neuroscience. 2018;33: 99-117. <http://doi.org/10.1016/j.dcn.2017.11.007>
2. T. A. Salthouse, C. Habeck, Q. Razlighi, et al. Breadth and age-dependency of relations between cortical thickness and cognition. Neurobiology of aging. 2015;36(11):3020-3028. <http://doi.org/10.1016/j.neurobiolaging.2015.08.011>
3. Y. Stern, C. Habeck, J. Steffener, et al. The Reference Ability Neural Network Study: motivation, design, and initial feasibility analyses. NeuroImage. 2014;103:139-151. <http://doi.org/10.1016/j.neuroimage.2014.09.029>
4. Y. Rosseel. lavaan: An R Package for Structural Equation Modeling. Journal of Statistical Software, 2012;48(2):1-36. <http://doi.org/10.18637/jss.v048.i02>

Supplementary Method Table1. Fit statistics of the latent change score.

|  | CFI | TLI | BIC | RMSEA |
| --- | --- | --- | --- | --- |
| fit | 0.846 | 0.843 | 23069.39 | 0.069, 95% CI: 0.065 -0.072, p<0.0001 |

Supplementary Method Table2. Full output of the latent change score model.

| lavaan 0.6-7 ended normally after 110 iterations    Estimator ML  Optimization method NLMINB  Number of free parameters 188  Number of equality constraints 68    Number of observations 254  Number of missing patterns 89    Model Test User Model:    Test statistic 2419.840  Degrees of freedom 1104  P-value (Chi-square) 0.000    Parameter Estimates:    Standard errors Standard  Information Observed  Observed information based on Hessian    Latent Variables:  Estimate Std.Err z-value P(>\|z\|)  slat0 =~  WAISRRAW_ (S1) 1.000  TMTAtm_bl (S2) -0.883 0.071 -12.482 0.000  STRPcRAW_ (S3) 0.925 0.066 13.999 0.000  DgtS_CRT_ (S4) -1.155 0.075 -15.339 0.000  PttC_CRT_ (S5) -0.909 0.078 -11.727 0.000  LtCm_CRT_ (S6) -0.961 0.077 -12.462 0.000  slat1 =~  WAISRRAW_ (S1) 1.000  TMTAtim_f (S2) -0.883 0.071 -12.482 0.000  STRPcRAW_ (S3) 0.925 0.066 13.999 0.000  DgtS_CRT_ (S4) -1.155 0.075 -15.339 0.000  PttC_CRT_ (S5) -0.909 0.078 -11.727 0.000  LtCm_CRT_ (S6) -0.961 0.077 -12.462 0.000  flat0 =~  WAIS3RAW_ (R1) 1.000  BLKraw_bl (R2) 0.956 0.062 15.343 0.000  WAIS3RAW_ (R3) 0.703 0.063 11.153 0.000  MtR_POTC_ (R4) 0.956 0.064 14.962 0.000  PpF_POTC_ (R5) 0.967 0.068 14.282 0.000  LtS_POTC_ (R6) 0.925 0.072 12.931 0.000  flat1 =~  WAIS3RAW_ (R1) 1.000  BLKraw_fu (R2) 0.956 0.062 15.343 0.000  WAIS3RAW_ (R3) 0.703 0.063 11.153 0.000  MtR_POTC_ (R4) 0.956 0.064 14.962 0.000  PpF_POTC_ (R5) 0.967 0.068 14.282 0.000  LtS_POTC_ (R6) 0.925 0.072 12.931 0.000  mlat0 =~  SRTlts_bl (M1) 1.000  SRTcltr_b (M2) 1.024 0.028 36.008 0.000  SRTlst_bl (M3) 0.962 0.036 26.942 0.000  LgM_POTC_ (M4) 0.373 0.059 6.282 0.000  PrA_POTC_ (M5) 0.372 0.060 6.152 0.000  WrO_POTC_ (M6) 0.435 0.057 7.660 0.000  mlat1 =~  SRTlts_fu (M1) 1.000  SRTcltr_f (M2) 1.024 0.028 36.008 0.000  SRTlast_f (M3) 0.962 0.036 26.942 0.000  LgM_POTC_ (M4) 0.373 0.059 6.282 0.000  PrA_POTC_ (M5) 0.372 0.060 6.152 0.000  WrO_POTC_ (M6) 0.435 0.057 7.660 0.000  vlat0 =~  WAISRRAW_ (V1) 1.000  WTARrw_bl (V2) 1.150 0.054 21.489 0.000  AMNARTrr_ (V3) -1.098 0.055 -19.981 0.000  Syn_POTC_ (V4) 0.978 0.041 23.673 0.000  Ant_POTC_ (V5) 1.086 0.055 19.816 0.000  PctNm_Pr_ (V6) -1.058 0.056 -18.985 0.000  vlat1 =~  Syn_POTC_ (V1) 1.000  Ant_POTC_ (V2) 1.150 0.054 21.489 0.000  PctNm_Pr_ (V3) -1.098 0.055 -19.981 0.000  WAISRRAW_ (V4) 0.978 0.041 23.673 0.000  WTARraw_f (V5) 1.086 0.055 19.816 0.000  AMNARTrr_ (V6) -1.058 0.056 -18.985 0.000  flcs =~  flat1 1.000  slcs =~  slat1 1.000  mlcs =~  mlat1 1.000  vlcs =~  vlat1 1.000    Regressions:  Estimate Std.Err z-value P(>\|z\|)  flat1 ~  flat0 1.000  flcs ~  flat0 0.018 0.036 0.508 0.611  slat1 ~  slat0 1.000  slcs ~  slat0 -0.004 0.038 -0.101 0.919  mlat1 ~  mlat0 1.000  mlcs ~  mlat0 -0.264 0.051 -5.167 0.000  vlat1 ~  vlat0 1.000  vlcs ~  vlat0 -0.073 0.032 -2.278 0.023    Covariances:  Estimate Std.Err z-value P(>\|z\|)  .WAISRdgtsymRAW_bl ~~  .WAISRRA (c_S1) 0.213 0.036 5.894 0.000  .TMTAtime_bl ~~  .TMTAtm_ (c_S2) 0.288 0.055 5.280 0.000  .STRPcRAW_bl ~~  .STRPRAW (c_S3) 0.278 0.044 6.349 0.000  .DgtSym_medianCorRT_bl ~~  .DS_CRT_ (c_S4) 0.143 0.038 3.787 0.000  .PattComp_medianCorRT_bl ~~  .PC_CRT_ (c_S5) 0.261 0.049 5.314 0.000  .LetComp_medianCorRT_bl ~~  .LC_CRT_ (c_S6) 0.161 0.048 3.392 0.001  .WAIS3matRAW_bl ~~  .WAIS3RA (c_R1) 0.164 0.036 4.554 0.000  .BLKraw_bl ~~  .BLKrw_f (c_R2) 0.241 0.037 6.588 0.000  .WAIS3letnumRAW_bl ~~  .WAIS3RA (c_R3) 0.266 0.046 5.790 0.000  .MatReas_PropOnTimeCor_bl ~~  .MR_POTC (c_R4) -0.011 0.035 -0.302 0.763  .PaperFold_PropOnTimeCor_bl ~~  .PF_POTC (c_R5) 0.065 0.039 1.677 0.094  .LetSet_PropOnTimeCor_bl ~~  .LS_POTC (c_R6) 0.104 0.048 2.193 0.028  .SRTlts_bl ~~  .SRTlts_ (c_M1) 0.039 0.017 2.298 0.022  .SRTcltr_bl ~~  .SRTclt_ (c_M2) -0.001 0.012 -0.073 0.941  .SRTlast_bl ~~  .SRTlst_ (c_M3) 0.046 0.023 2.027 0.043  .LogMem_PropOnTimeCor_bl ~~  .LM_POTC (c_M4) 0.230 0.069 3.317 0.001  .PairAssoc_PropOnTimeCor_bl ~~  .PA_POTC (c_M5) 0.357 0.072 4.982 0.000  .WordOrder_PropOnTimeCor_bl ~~  .WO_POTC (c_M6) 0.294 0.065 4.534 0.000  .WAISRvocRAW_bl ~~  .WAISRRA (c_V1) 0.151 0.026 5.878 0.000  .WTARraw_bl ~~  .WTARrw_ (c_V2) 0.033 0.014 2.345 0.019  .AMNARTerr_bl ~~  .AMNART_ (c_V3) -0.005 0.015 -0.293 0.770  .Syn_PropOnTimeCor_bl ~~  .S_POTC_ (c_V4) 0.138 0.035 3.951 0.000  .Ant_PropOnTimeCor_bl ~~  .A_POTC_ (c_V5) 0.126 0.048 2.644 0.008  .PictName_Prop_bl ~~  .PctN_P_ (c_V6) 2.298 0.250 9.195 0.000  slat0 ~~  flat0 0.390 0.055 7.140 0.000  mlat0 0.379 0.057 6.620 0.000  vlat0 0.137 0.044 3.136 0.002  flat0 ~~  mlat0 0.371 0.057 6.465 0.000  vlat0 0.329 0.051 6.493 0.000  mlat0 ~~  vlat0 0.125 0.049 2.532 0.011  .flcs ~~  .slcs 0.043 0.011 3.883 0.000  .mlcs 0.049 0.019 2.569 0.010  .vlcs 0.032 0.009 3.414 0.001  .slcs ~~  .mlcs 0.078 0.020 3.830 0.000  .vlcs 0.026 0.009 2.803 0.005  .mlcs ~~  .vlcs 0.061 0.016 3.686 0.000    Intercepts:  Estimate Std.Err z-value P(>\|z\|)  .WAISRR (b0_S1) 0.035 0.060 0.582 0.561  .WAISRR (b0_S1) 0.035 0.060 0.582 0.561  .TMTAt_ (b0_S2) 0.059 0.063 0.931 0.352  .TMTAt_ (b0_S2) 0.059 0.063 0.931 0.352  .STRPRA (b0_S3) -0.036 0.061 -0.594 0.553  .STRPRA (b0_S3) -0.036 0.061 -0.594 0.553  .DS_CRT (b0_S4) -0.100 0.067 -1.482 0.138  .DS_CRT (b0_S4) -0.100 0.067 -1.482 0.138  .PC_CRT (b0_S5) -0.025 0.065 -0.390 0.697  .PC_CRT (b0_S5) -0.025 0.065 -0.390 0.697  .LC_CRT (b0_S6) -0.032 0.064 -0.498 0.619  .LC_CRT (b0_S6) -0.032 0.064 -0.498 0.619  .WAIS3R (b0_R1) 0.034 0.063 0.542 0.588  .WAIS3R (b0_R1) 0.034 0.063 0.542 0.588  .BLKrw_ (b0_R2) -0.014 0.062 -0.228 0.820  .BLKrw_ (b0_R2) -0.014 0.062 -0.228 0.820  .WAIS3R (b0_R3) -0.094 0.056 -1.660 0.097  .WAIS3R (b0_R3) -0.094 0.056 -1.660 0.097  .MR_POT (b0_R4) 0.091 0.060 1.527 0.127  .MR_POT (b0_R4) 0.091 0.060 1.527 0.127  .PF_POT (b0_R5) 0.056 0.063 0.892 0.372  .PF_POT (b0_R5) 0.056 0.063 0.892 0.372  .LS_POT (b0_R6) 0.024 0.064 0.369 0.712  .LS_POT (b0_R6) 0.024 0.064 0.369 0.712  .SRTlt_ (b0_M1) -0.009 0.063 -0.140 0.889  .SRTlt_ (b0_M1) -0.009 0.063 -0.140 0.889  .SRTcl_ (b0_M2) 0.011 0.061 0.181 0.856  .SRTcl_ (b0_M2) 0.011 0.061 0.181 0.856  .SRTls_ (b0_M3) -0.008 0.063 -0.123 0.902  .SRTls_ (b0_M3) -0.008 0.063 -0.123 0.902  .LM_POT (b0_M4) -0.007 0.058 -0.116 0.908  .LM_POT (b0_M4) -0.007 0.058 -0.116 0.908  .PA_POT (b0_M5) 0.018 0.060 0.306 0.759  .PA_POT (b0_M5) 0.018 0.060 0.306 0.759  .WO_POT (b0_M6) 0.072 0.058 1.240 0.215  .WO_POT (b0_M6) 0.072 0.058 1.240 0.215  .WAISRR (b0_V1) 0.013 0.066 0.192 0.848  .WAISRR (b0_V1) 0.013 0.066 0.192 0.848  .WTARr_ (b0_V2) 0.089 0.068 1.313 0.189  .WTARr_ (b0_V2) 0.089 0.068 1.313 0.189  .AMNART (b0_V3) -0.084 0.066 -1.273 0.203  .AMNART (b0_V3) -0.084 0.066 -1.273 0.203  .S_POTC (b0_V4) -0.019 0.069 -0.282 0.778  .S_POTC (b0_V4) -0.019 0.069 -0.282 0.778  .A_POTC (b0_V5) -0.104 0.078 -1.338 0.181  .A_POTC (b0_V5) -0.104 0.078 -1.338 0.181  .PcN_P_ (b0_V6) 0.109 0.126 0.872 0.383  .PcN_P_ (b0_V6) 0.109 0.126 0.872 0.383  .flcs -0.148 0.027 -5.509 0.000  .slcs -0.174 0.027 -6.526 0.000  .mlcs -0.110 0.049 -2.241 0.025  .vlcs 0.066 0.024 2.708 0.007  slat0 0.000  .slat1 0.000  flat0 0.000  .flat1 0.000  mlat0 0.000  .mlat1 0.000  vlat0 0.000  .vlat1 0.000    Variances:  Estimate Std.Err z-value P(>\|z\|)  .WAISRRA (v_S1) 0.384 0.036 10.620 0.000  .TMTAtm_ (v_S2) 0.724 0.055 13.158 0.000  .STRPRAW (v_S3) 0.524 0.044 11.989 0.000  .DS_CRT_ (v_S4) 0.292 0.036 8.073 0.000  .PC_CRT_ (v_S5) 0.522 0.049 10.750 0.000  .LC_CRT_ (v_S6) 0.491 0.046 10.776 0.000  .WAISRRA (v_S1) 0.384 0.036 10.620 0.000  .TMTAtm_ (v_S2) 0.724 0.055 13.158 0.000  .STRPRAW (v_S3) 0.524 0.044 11.989 0.000  .DS_CRT_ (v_S4) 0.292 0.036 8.073 0.000  .PC_CRT_ (v_S5) 0.522 0.049 10.750 0.000  .LC_CRT_ (v_S6) 0.491 0.046 10.776 0.000  .WAIS3RA (v_R1) 0.435 0.036 12.002 0.000  .BLKrw_b (v_R2) 0.406 0.037 11.112 0.000  .WAIS3RA (v_R3) 0.626 0.046 13.621 0.000  .MR_POTC (v_R4) 0.372 0.033 11.323 0.000  .PF_POTC (v_R5) 0.436 0.038 11.523 0.000  .LS_POTC (v_R6) 0.544 0.046 11.932 0.000  .WAIS3RA (v_R1) 0.435 0.036 12.002 0.000  .BLKrw_f (v_R2) 0.406 0.037 11.112 0.000  .WAIS3RA (v_R3) 0.626 0.046 13.621 0.000  .MR_POTC (v_R4) 0.372 0.033 11.323 0.000  .PF_POTC (v_R5) 0.436 0.038 11.523 0.000  .LS_POTC (v_R6) 0.544 0.046 11.932 0.000  .SRTlts_ (v_M1) 0.200 0.018 11.417 0.000  .SRTclt_ (v_M2) 0.070 0.013 5.237 0.000  .SRTlst_ (v_M3) 0.308 0.023 13.345 0.000  .LM_POTC (v_M4) 0.866 0.066 13.102 0.000  .PA_POTC (v_M5) 0.864 0.069 12.570 0.000  .WO_POTC (v_M6) 0.769 0.061 12.605 0.000  .SRTlts_ (v_M1) 0.200 0.018 11.417 0.000  .SRTclt_ (v_M2) 0.070 0.013 5.237 0.000  .SRTlst_ (v_M3) 0.308 0.023 13.345 0.000  .LM_POTC (v_M4) 0.866 0.066 13.102 0.000  .PA_POTC (v_M5) 0.864 0.069 12.570 0.000  .WO_POTC (v_M6) 0.769 0.061 12.605 0.000  .WAISRRA (v_V1) 0.317 0.026 12.404 0.000  .WTARrw_ (v_V2) 0.129 0.014 9.015 0.000  .AMNART_ (v_V3) 0.166 0.016 10.419 0.000  .S_POTC_ (v_V4) 0.379 0.033 11.414 0.000  .A_POTC_ (v_V5) 0.560 0.046 12.066 0.000  .PctN_P_ (v_V6) 2.551 0.250 10.206 0.000  .WAISRRA (v_V1) 0.317 0.026 12.404 0.000  .WTARrw_ (v_V2) 0.129 0.014 9.015 0.000  .AMNART_ (v_V3) 0.166 0.016 10.419 0.000  .S_POTC_ (v_V4) 0.379 0.033 11.414 0.000  .A_POTC_ (v_V5) 0.560 0.046 12.066 0.000  .PctN_P_ (v_V6) 2.551 0.250 10.206 0.000  slat0 0.590 0.078 7.595 0.000  .slat1 0.000  flat0 0.616 0.080 7.682 0.000  .flat1 0.000  mlat0 0.850 0.087 9.823 0.000  .mlat1 0.000  vlat0 0.633 0.074 8.500 0.000  .vlat1 0.000  .flcs 0.035 0.016 2.211 0.027  .slcs 0.066 0.017 3.980 0.000  .mlcs 0.463 0.052 8.981 0.000  .vlcs 0.061 0.014 4.504 0.000 |
| --- |

Supplementary Method Figure1. Latent change score model.


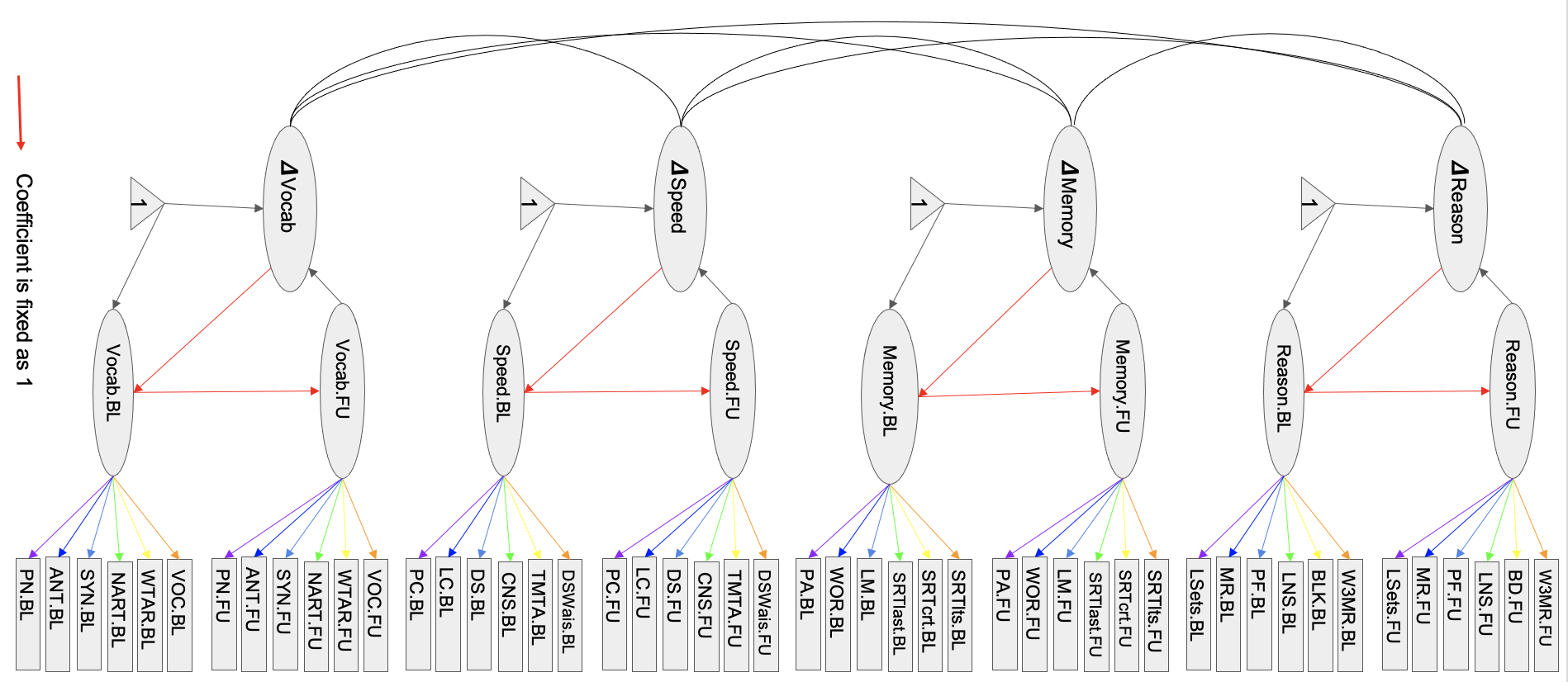


# sTable 1. Comparison of the participants’ characteristics between those who were included in the final models and who were not, baseline.

|  | Participants included in the analysis (n=198) | Participants *not* included in the analysis (n=364) | p-diff |
| --- | --- | --- | --- |
| Age, mean (SD), years | 54.38 (16.42) | 53.01 (17.40) | 0.362 |
| Sex |  |  |  |
| Male, n (%) | 90 (45.45) | 153 (42.03) | 0.434 |
| Female, n (%) | 108 (54.55) | 211 (57.97) |  |
| Education^a^, mean (SD), years | 16.34 (2.42) | 16.06 (2.34) | 0.176 |
| Race/ethnicity^b^ |  |  |  |
| Non-Hispanic White, n (%) | 119 (60.10) | 192 (53.04) | 0.220 |
| Non-Hispanic Black, n (%) | 46 (23.23) | 87 (24.03) |  |
| Others, n (%) | 10 (5.05) | 33 (9.12) |  |
| Hispanic, n (%) | 23 (11.62) | 50 (13.81) |  |

^a^ Three missing values are included in the “participants not included in the analysis” group.

^b^ Two missing values are included in the “participants not included in the analysis” group.

# sTable 2. Spearman’s correlation between white matter hyperintensity (WMH) and cognitive change within leisure time physical activity (LTPA) and occupational physical activity (OPA) groups (n=198).

|  |  | Total population (n=198) | | | Aged ≥43 years (n=147) | | | Aged <43 years (n=51) | | | |
| --- | --- | --- | --- | --- | --- | --- | --- | --- | --- | --- | --- |
|  |  | Low LTPA (n=99) | High LTPA (n=99) | p-diff^a^ | Low LTPA (n=77) | High LTPA (n=70) | p-diff^a^ | | Low LTPA (n=22) | High LTPA (n=29) | p-diff^a^ |
| Global cognition change | rho | -0.166 | **-0.278**** | 0.414 | -0.129 | -0.117 | 0.942 | | 0.051 | -0.182 | 0.436 |
|  | *p* | 0.100 | **0.005** |  | 0.262 | 0.336 |  | | 0.820 | 0.344 |  |
| Fluid reasoning change | rho | **-0.360***** | **-0.448***** | 0.466 | **-0.313**** | -0.190 | 0.435 | | 0.110 | -0.215 | 0.276 |
|  | *p* | **<0.001** | **<0.001** |  | **0.006** | 0.114 |  | | 0.626 | 0.262 |  |
| Processing speed change | rho | **-0.299**** | **-0.329***** | 0.818 | **-0.253*** | -0.139 | 0.842 | | 0.212 | -0.148 | 0.227 |
|  | *p* | **0.003** | **0.001** |  | **0.027** | 0.253 |  | | 0.344 | 0.444 |  |
| Memory change | rho | -0.079 | -0.134 | 0.700 | -0.092 | -0.089 | 0.986 | | -0.046 | -0.079 | 0.913 |
|  | *p* | 0.434 | 0.187 |  | 0.428 | 0.465 |  | | 0.840 | 0.683 |  |
| Vocabulary change | rho | **-0.266**** | **-0.362***** | 0.460 | -0.123 | -0.071 | 0.756 | | 0.112 | -0.280 | 0.185 |
|  | *p* | **0.008** | **<0.001** |  | 0.285 | 0.557 |  | | 0.619 | 0.142 |  |
|  |  | Low OPA (n=99) | High OPA (n=99) |  | Low OPA (n=72) | High OPA (n=75) |  | | Low OPA (n=27) | High OPA (n=24) |  |
| Global cognition change | rho | **-0.283**** | -0.164 | 0.385 | -0.166 | -0.059 | 0.520 | | -0.261 | 0.053 | 0.284 |
|  | *p* | **0.005** | 0.105 |  | 0.162 | 0.612 |  | | 0.189 | 0.806 |  |
| Fluid reasoning change | rho | **-0.461***** | **-0.345***** | 0.336 | **-0.250*** | -0.213 | 0.817 | | -0.253 | 0.099 | 0.231 |
|  | *p* | **<0.001** | **<0.001** |  | **0.034** | 0.067 |  | | 0.203 | 0.645 |  |
| Processing speed change | rho | **-0.383***** | **-0.262**** | 0.349 | **-0.247*** | -0.144 | 0.525 | | -0.107 | 0.137 | 0.412 |
|  | *p* | **<0.001** | **0.009** |  | **0.036** | 0.218 |  | | 0.596 | 0.522 |  |
| Memory change | rho | -0.148 | -0.082 | 0.643 | -0.140 | -0.057 | 0.619 | | -0.239 | 0.043 | 0.337 |
|  | *p* | 0.143 | 0.417 |  | 0.240 | 0.630 |  | | 0.230 | 0.840 |  |
| Vocabulary change | rho | **-0.408***** | -0.197 | 0.106 | -0.111 | -0.049 | 0.711 | | -0.237 | -0.026 | 0.471 |
|  | *p* | **<0.001** | 0.050 |  | 0.354 | 0.677 |  | | 0.234 | 0.904 |  |

*p<0.05; **p<0.01; ***p<0.001.

Abbreviation: LTPA=Leisure time physical activity; OPA=Occupational physical activity; WMH=White matter hyperintensity.

^a^ p-diff values referred to the significance between low- versus high-PA group, which were calculated in MedCalc (Free trial) software. <https://www.medcalc.org/>

The dichotomous PA groups were split by median PA.

# sTable 3. The interaction of dichotomous physical activity group with white matter hyperintensity (WMH) on cognitive change.

|  |  | Global cognition change | | | Fluid reasoning change | | | Processing speed change | | | Memory change | | | Vocabulary change | | |
| --- | --- | --- | --- | --- | --- | --- | --- | --- | --- | --- | --- | --- | --- | --- | --- | --- |
|  |  | Model 1 | Model 2 | Model 3 | Model 1 | Model 2 | Model 3 | Model 1 | Model 2 | Model 3 | Model 1 | Model 2 | Model 3 | Model 1 | Model 2 | Model 3 |
| Total population | |  |  |  |  |  |  |  |  |  |  |  |  |  |  |  |
| TPA*WMH | Estimate | 0.032 | 0.028 | 0.032 | **0.037**** | **0.029**** | **0.033**** | **0.042**** | **0.037*** | **0.039**** | 0.047 | 0.053 | 0.049 | **0.051***** | **0.044***** | **0.048***** |
|  | 95% CI | -0.022 - 0.087 | -0.024 - 0.080 | -0.024 - 0.087 | **0.009 - 0.065** | **0.004 - 0.054** | **0.006 - 0.060** | **0.004 - 0.080** | **-3.48e-05 - 0.073** | **0.0005 - 0.078** | -0.098 - 0.193 | -0.095 - 0.200 | -0.106 - 0.205 | **0.014 - 0.089** | **0.014 - 0.075** | **0.015 - 0.081** |
|  | *p* | 0.245 | 0.295 | 0.260 | **0.010** | **0.024** | **0.018** | **0.030** | **0.050** | **0.047** | 0.520 | 0.481 | 0.533 | **0.008** | **0.005** | **0.005** |
| LTPA*WMH | Estimate | -0.003 | -0.015 | -0.010 | -0.001 | -0.012 | -0.009 | -0.002 | -0.013 | -0.010 | 0.013 | -0.005 | 0.001 | 0.008 | -0.003 | 0.003 |
|  | 95% CI | -0.058 - 0.052 | -0.067 - 0.038 | -0.065 - 0.044 | -0.029 - 0.027 | -0.037 - 0.013 | -0.035 - 0.018 | -0.040 - 0.036 | -0.050 - 0.024 | -0.049 - 0.029 | -0.131 - 0.158 | -0.151 - 0.140 | -0.152 - 0.154 | -0.030 - 0.047 | -0.034 - 0.028 | -0.030 - 0.036 |
|  | *p* | 0.926 | 0.583 | 0.712 | 0.936 | 0.355 | 0.520 | 0.918 | 0.476 | 0.604 | 0.856 | 0.941 | 0.992 | 0.667 | 0.847 | 0.849 |
| OPA*WMH | Estimate | 0.029 | 0.019 | 0.024 | 0.021 | 0.011 | 0.014 | 0.024 | 0.016 | 0.020 | 0.060 | 0.053 | 0.060 | **0.034*** | 0.025 | 0.025 |
|  | 95% CI | -0.026 - 0.084 | -0.033 - 0.071 | -0.031 - 0.079 | -0.007 - 0.049 | -0.014 - 0.036 | -0.013 - 0.041 | -0.014 - 0.062 | -0.021 - 0.052 | -0.019 - 0.059 | -0.085 - 0.205 | -0.092 - 0.198 | -0.094 - 0.214 | **-0.004 - 0.072** | -0.0062- 0.056 | -0.009 - 0.058 |
|  | *p* | 0.303 | 0.468 | 0.386 | 0.145 | 0.378 | 0.305 | 0.218 | 0.402 | 0.317 | 0.418 | 0.471 | 0.441 | **0.078** | 0.108 | 0.144 |
| Aged ≥ 43 years | |  |  |  |  |  |  |  |  |  |  |  |  |  |  |  |
| TPA*WMH | Estimate | 0.060 | 0.051 | 0.043 | **0.059***** | **0.046***** | **0.048***** | **0.066**** | **0.057**** | **0.054**** | 0.081 | 0.077 | 0.041 | **0.065**** | **0.053***** | **0.057***** |
|  | 95% CI | -0.017 - 0.137 | -0.021 - 0.124 | -0.032 - 0.119 | **0.021 - 0.096** | **0.015 - 0.077** | **0.015 - 0.081** | **0.016 - 0.115** | **0.010 - 0.104** | **0.004 - 0.103** | -0.121 - 0.283 | -0.129 - 0.284 | -0.176 - 0.257 | **0.015 - 0.116** | **0.013 - 0.092** | **0.015 - 0.100** |
|  | *p* | 0.124 | 0.163 | 0.259 | **0.002** | **0.004** | **0.005** | **0.010** | **0.018** | **0.033** | 0.428 | 0.460 | 0.711 | **0.012** | **0.009** | **0.008** |
| LTPA*WMH | Estimate | 0.018 | 0.003 | 0.005 | 0.013 | -0.001 | -0.001 | 0.007 | -0.007 | -0.008 | 0.017 | 0.004 | 0.012 | 0.025 | 0.007 | 0.008 |
|  | 95% CI | -0.060 - 0.096 | -0.070 - 0.076 | -0.070 - 0.081 | -0.026 - 0.052 | -0.033 - 0.031 | -0.035 - 0.033 | -0.044- 0.058 | -0.055 - 0.041 | -0.059 - 0.042 | -0.184 - 0.219 | -0.203 - 0.210 | -0.203 - 0.226 | -0.027 - 0.078 | -0.034 - 0.0475 | -0.035 - 0.052 |
|  | *p* | 0.651 | 0.940 | 0.887 | 0.514 | 0.941 | 0.958 | 0.778 | 0.768 | 0.747 | 0.864 | 0.971 | 0.915 | 0.342 | 0.741 | 0.704 |
| OPA*WMH | Estimate | 0.049 | 0.029 | 0.018 | 0.024 | 0.011 | 0.009 | 0.033 | 0.021 | 0.019 | 0.156 | 0.121 | 0.093 | 0.027 | 0.012 | 0.011 |
|  | 95% CI | -0.029 - 0.127 | -0.042 - 0.101 | -0.058 - 0.095 | -0.014 - 0.063 | -0.021 - 0.042 | -0.025 - 0.043 | -0.018 - 0.084 | -0.025 - 0.068 | -0.031 - 0.069 | -0.047 - 0.358 | -0.083 - 0.324 | -0.126 - 0.311 | -0.025 - 0.079 | -0.028 - 0.052 | -0.033 - 0.054 |
|  | *p* | 0.215 | 0.420 | 0.634 | 0.213 | 0.511 | 0.601 | 0.201 | 0.367 | 0.459 | 0.131 | 0.243 | 0.403 | 0.310 | 0.561 | 0.624 |
| Aged < 43 years | |  |  |  |  |  |  |  |  |  |  |  |  |  |  |  |
| TPA*WMH | Estimate | -0.001 | 0.008 | -0.029 | -0.022 | 0.010 | -0.020 | -0.019 | -0.005 | -0.058 | 0.180 | 0.126 | 0.134 | -0.055 | 0.029 | 0.004 |
|  | 95% CI | -0.239 - 0.236 | -0.264 - 0.281 | -0.357 - 0.299 | -0.149 - 0.105 | -0.142 - 0.162 | -0.201 - 0.160 | -0.198 - 0.161 | -0.229 - 0.220 | -0.323 - 0.207 | -0.471 - 0.831 | -0.584 - 0.836 | -0.763 - 1.030 | -0.223 - 0.114 | -0.138 - 0.196 | -0.201 - 0.208 |
|  | *p* | 0.990 | 0.953 | 0.857 | 0.732 | 0.895 | 0.820 | 0.835 | 0.966 | 0.657 | 0.579 | 0.722 | 0.763 | 0.518 | 0.730 | 0.971 |
| LTPA*WMH | Estimate | -0.018 | 0.075 | 0.127 | 0.011 | 0.046 | 0.069 | 0.010 | 0.051 | 0.079 | -0.092 | 0.059 | 0.163 | -0.016 | 0.039 | 0.077 |
|  | 95% CI | -0.248 - 0.211 | -0.176 - 0.327 | -0.163 - 0.417 | -0.112 - 0.135 | -0.092 - 0.184 | -0.087 - 0.225 | -0.165 - 0.186 | -0.147 - 0.249 | -0.144 - 0.302 | -0.711 - 0.526 | -0.626 - 0.743 | -0.651 - 0.977 | -0.180 - 0.147 | -0.124 - 0.203 | -0.114 - 0.267 |
|  | *p* | 0.873 | 0.547 | 0.379 | 0.856 | 0.508 | 0.371 | 0.905 | 0.604 | 0.474 | 0.765 | 0.863 | 0.686 | 0.843 | 0.628 | 0.418 |
| OPA*WMH | Estimate | 0.095 | 0.052 | -0.005 | 0.019 | 0.025 | 0.002 | 0.007 | -0.011 | -0.054 | 0.288 | 0.101 | -0.019 | 0.055 | 0.086 | 0.070 |
|  | 95% CI | -0.137 - 0.327 | -0.212 - 0.316 | -0.323 - 0.312 | -0.106 - 0.144 | -0.118 - 0.168 | -0.169 - 0.173 | -0.170 - 0.183 | -0.220 - 0.198 | -0.297 - 0.188 | -0.333 - 0.908 | -0.588 - 0.789 | -0.884 - 0.846 | -0.109 - 0.220 | -0.068 - 0.240 | -0.123 - 0.262 |
|  | *p* | 0.414 | 0.691 | 0.973 | 0.757 | 0.727 | 0.981 | 0.940 | 0.916 | 0.652 | 0.355 | 0.769 | 0.965 | 0.503 | 0.264 | 0.464 |

*p<0.1; **p<0.05; ***p<0.01.

Abbreviation: TPA=Total physical activity; LTPA=Leisure time physical activity; OPA=Occupational physical activity; WMH=White matter hyperintensity; 95% CI=95% Confidence interval.

Covariates: Model 1: age, sex, education, baseline cognition. Model 2: Model 1+ IQ, race/ethnicity (categorical), cardiovascular risk index. Model 3: Model 2+ total brain volume, mean cortical thickness.

The dichotomous PA groups were split by median PA, and low PA group was used as the reference group. The two levels of LTPA were high (≥1260 MET-min/week) and low (0 to <1260 MET-min/week). People included in the high LTPA group should perform at least 2.33 hours of vigorous activities, 4.2 hours of moderate activities, or 7 hours of light activities per week. In terms of OPA, the people in high OPA group mainly work on jobs with high demands of outdoor and physical activities, such as construction workers, firefighters, police, truck drivers, real estate sales agents, etc.

# sFigure 1. The association between leisure time physical activity and occupational physical activity.


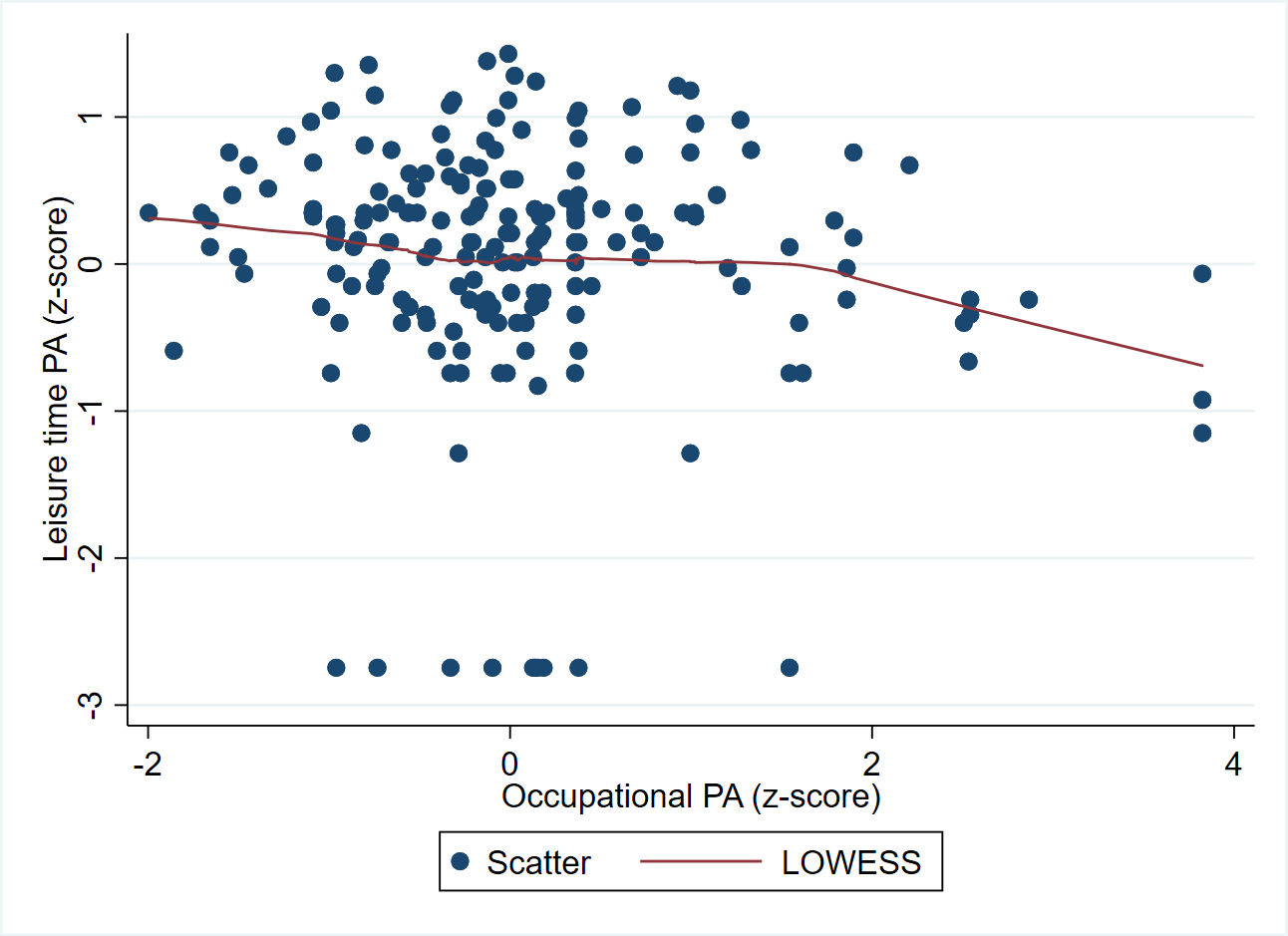


# sFigure 2. The relationship between white matter hyperintensity (WMH) and cognitive change by leisure time physical activity (LTPA) group.


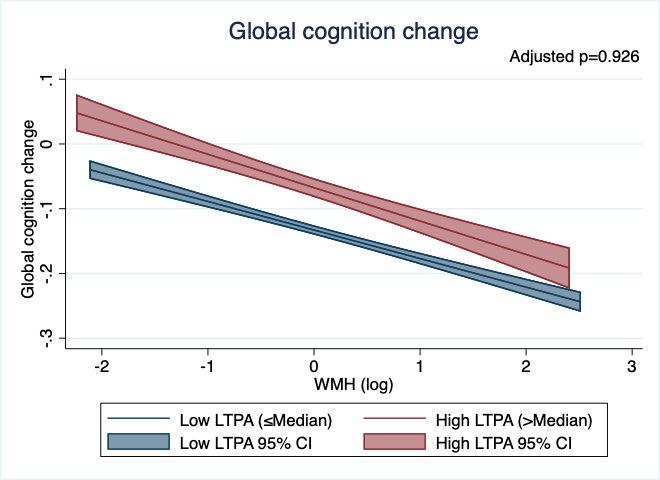

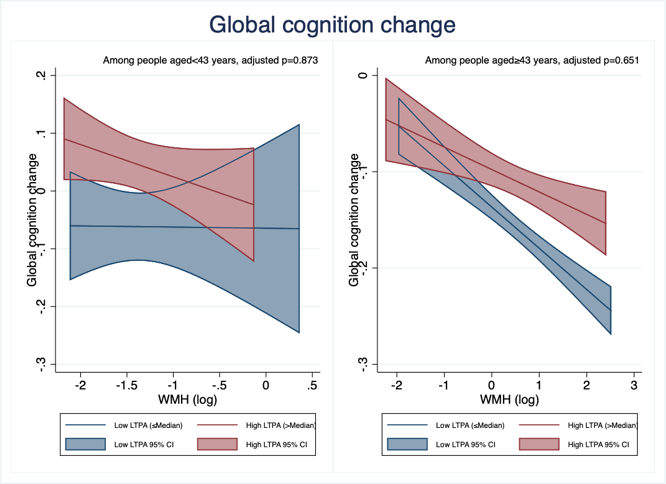

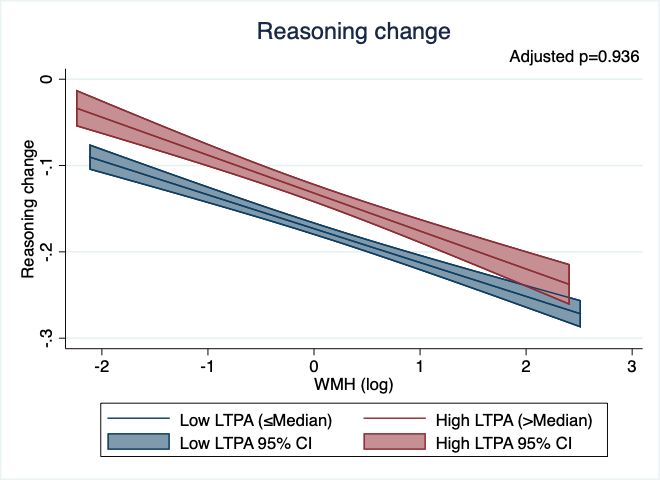

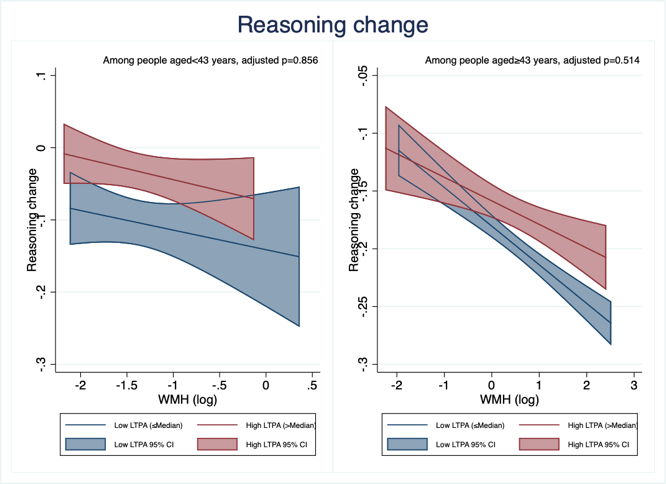

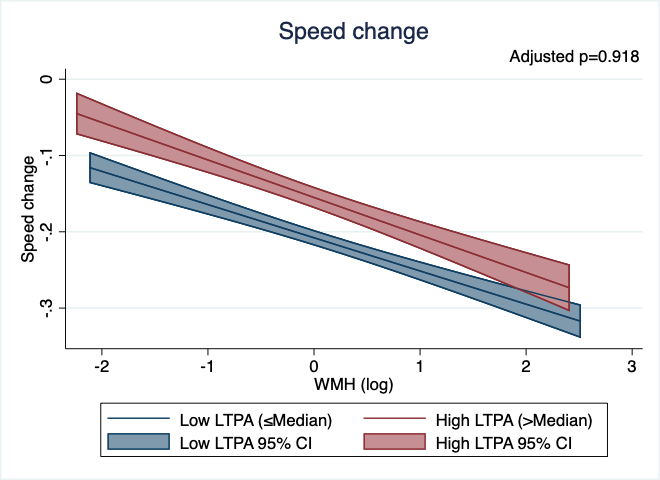

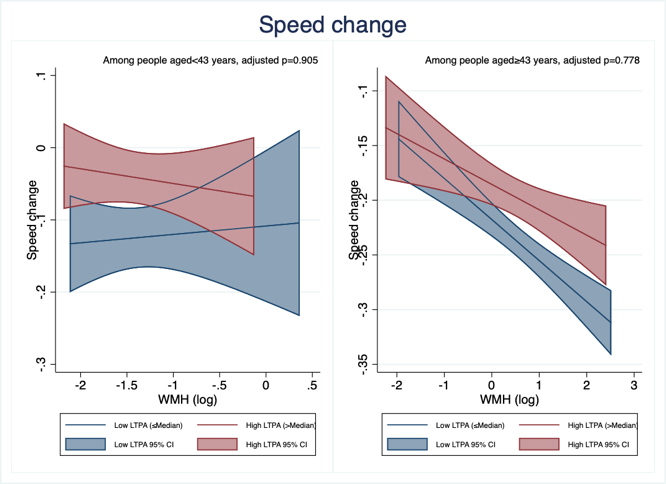


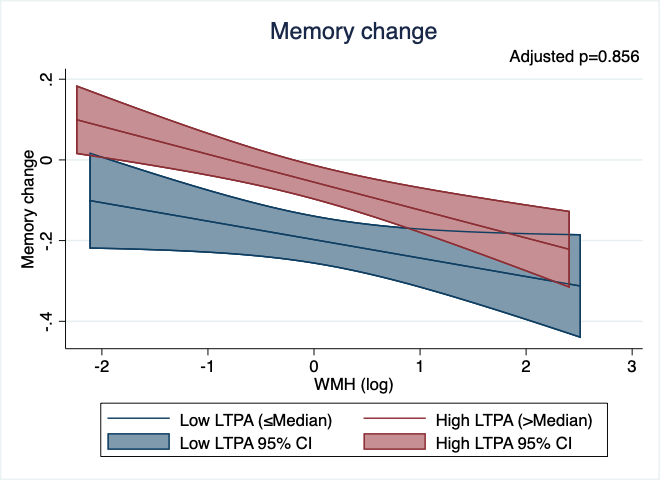

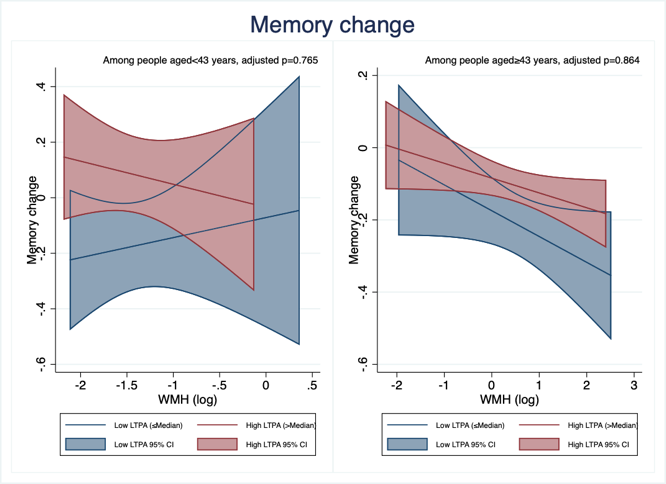


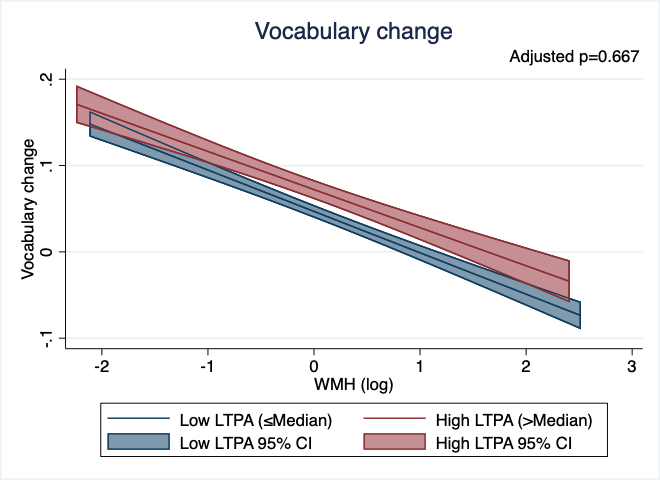

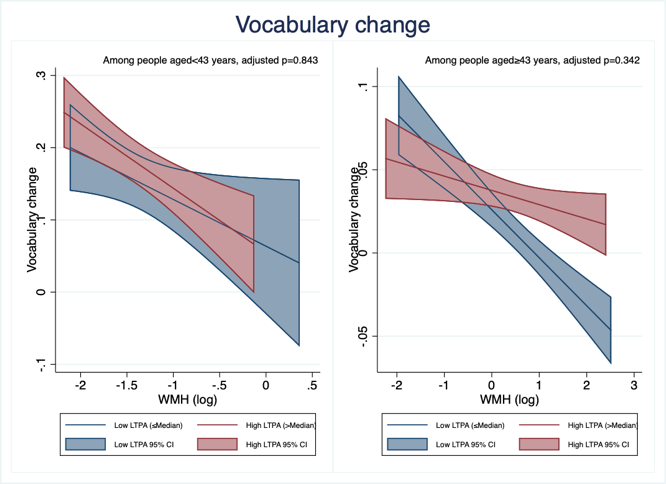


Abbreviation: LTPA=Leisure time physical activity; WMH=White matter hyperintensity.

All models controlled for age, sex, education and baseline cognition.

Adjusted p-values were the p-values of the interaction term between WMH and dichotomous LTPA group.

# sFigure 3. The relationship between white matter hyperintensity (WMH) and cognitive change by occupational physical activity (OPA) group.


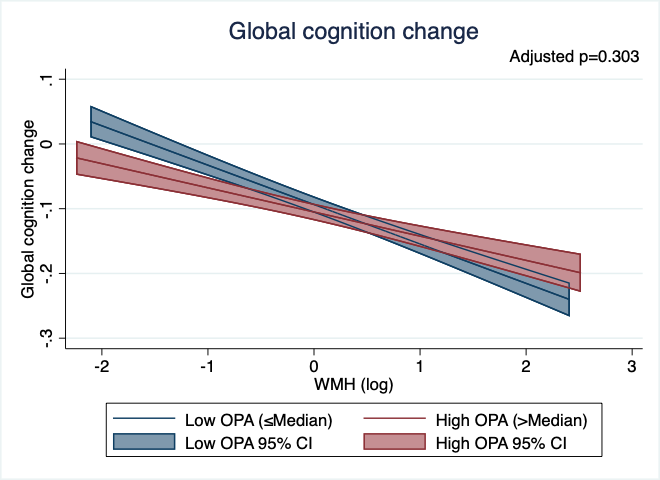

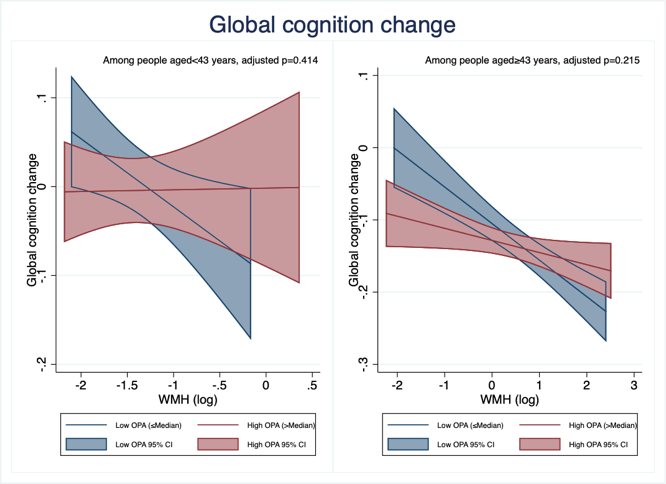

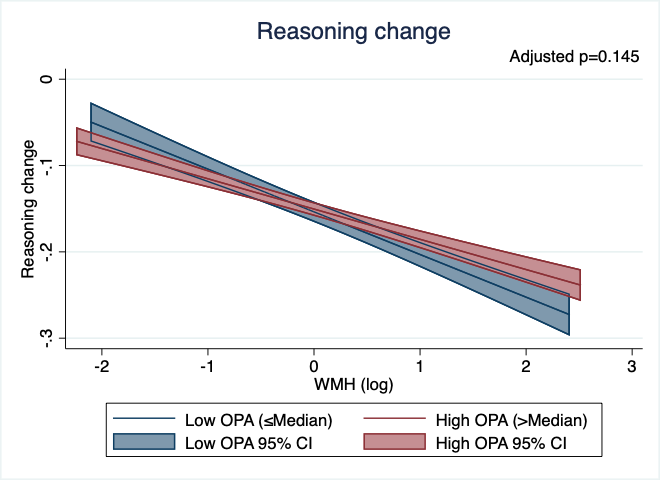

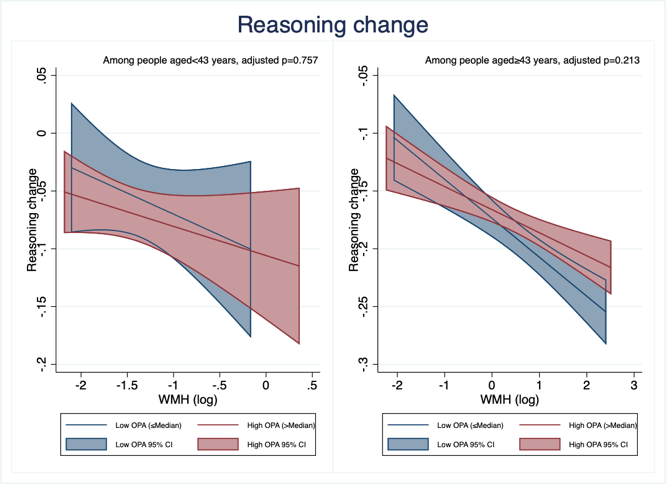

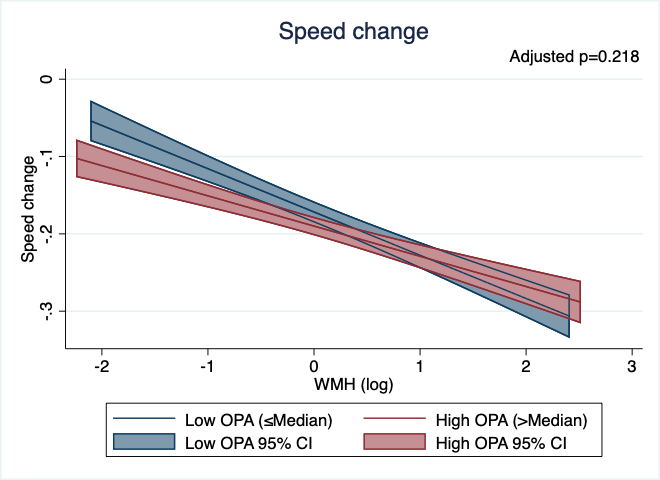

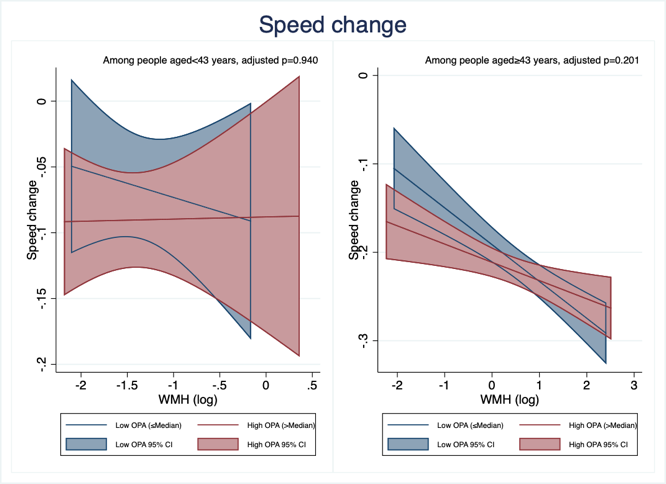

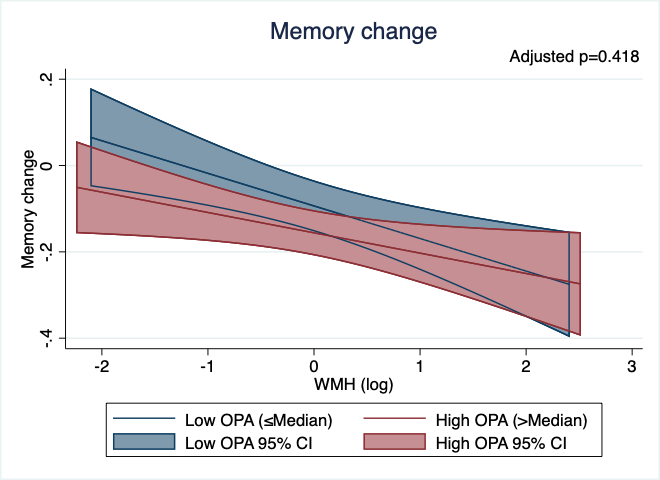

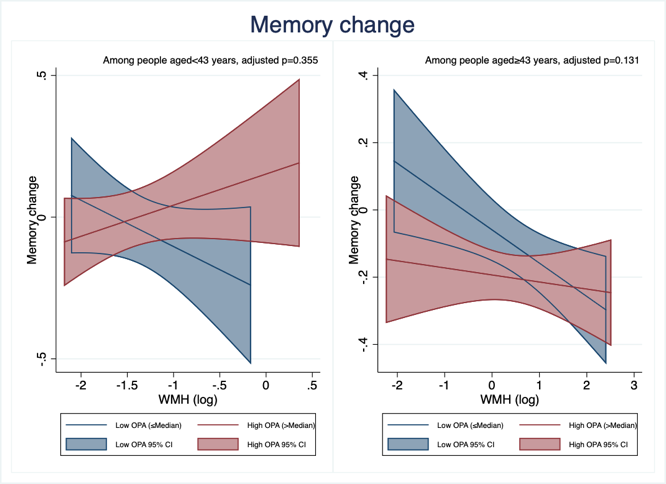


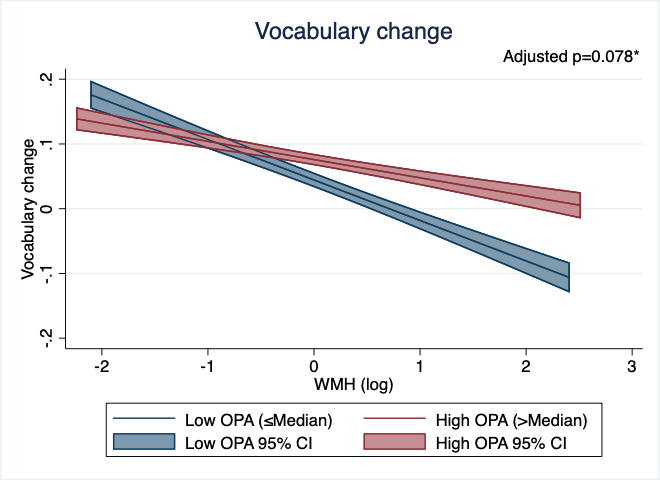

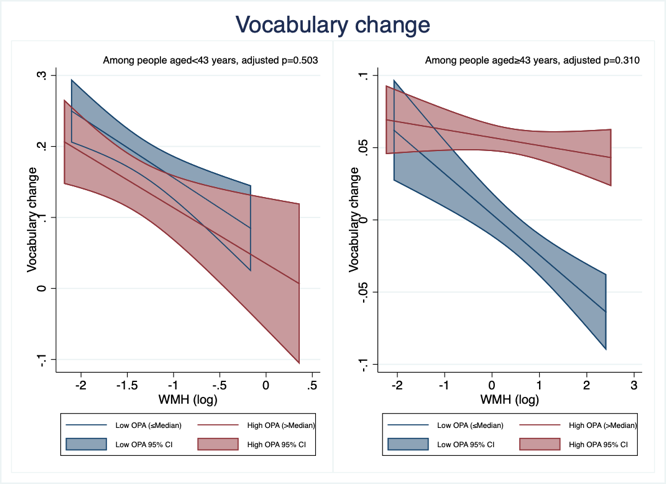


Abbreviation: OPA=Occupational physical activity; WMH=White matter hyperintensity.

All models controlled for age, sex, education and baseline cognition.

Adjusted p-values were the p-values of the interaction term between WMH and dichotomous OPA group.
